# Metabolism of PLTP, CETP, and LCAT on multiple HDL sizes using the Orbitrap Fusion Lumos

**DOI:** 10.1172/jci.insight.143526

**Published:** 2021-02-08

**Authors:** Sasha A. Singh, Allison B. Andraski, Hideyuki Higashi, Lang Ho Lee, Ashisha Ramsaroop, Frank M. Sacks, Masanori Aikawa

**Affiliations:** 1 Center for Interdisciplinary Cardiovascular Sciences, Department of Medicine, Brigham and Women’s Hospital, Harvard Medical School, Boston, Massachusetts, USA.; 2 Department of Nutrition and Department of Molecular Metabolism, Harvard T.H. Chan School of Public Health, Boston, Massachusetts, USA.; 3 Channing Division of Network Medicine, Department of Medicine, and; 4 Center for Excellence in Vascular Biology, Division of Cardiovascular Medicine, Department of Medicine, Brigham and Women’s Hospital, Harvard Medical School, Boston, Massachusetts, USA.

**Keywords:** Vascular Biology, Lipoproteins, Proteomics

## Abstract

Recent in vivo tracer studies demonstrated that targeted mass spectrometry (MS) on the Q Exactive Orbitrap could determine the metabolism of HDL proteins 100s-fold less abundant than apolipoprotein A1 (APOA1). In this study, we demonstrate that the Orbitrap Lumos can measure tracer in proteins whose abundances are 1000s-fold less than APOA1, specifically the lipid transfer proteins phospholipid transfer protein (PLTP), cholesterol ester transfer protein (CETP), and lecithin-cholesterol acyl transferase (LCAT). Relative to the Q Exactive, the Lumos improved tracer detection by reducing tracer enrichment compression, thereby providing consistent enrichment data across multiple HDL sizes from 6 participants. We determined by compartmental modeling that PLTP is secreted in medium and large HDL (alpha2, alpha1, and alpha0) and is transferred from medium to larger sizes during circulation from where it is catabolized. CETP is secreted mainly in alpha1 and alpha2 and remains in these sizes during circulation. LCAT is secreted mainly in medium and small HDL (alpha2, alpha3, prebeta). Unlike PLTP and CETP, LCAT’s appearance on HDL is markedly delayed, indicating that LCAT may reside for a time outside of systemic circulation before attaching to HDL in plasma. The determination of these lipid transfer proteins’ unique metabolic structures was possible due to advances in MS technologies.

## Graphical abstract

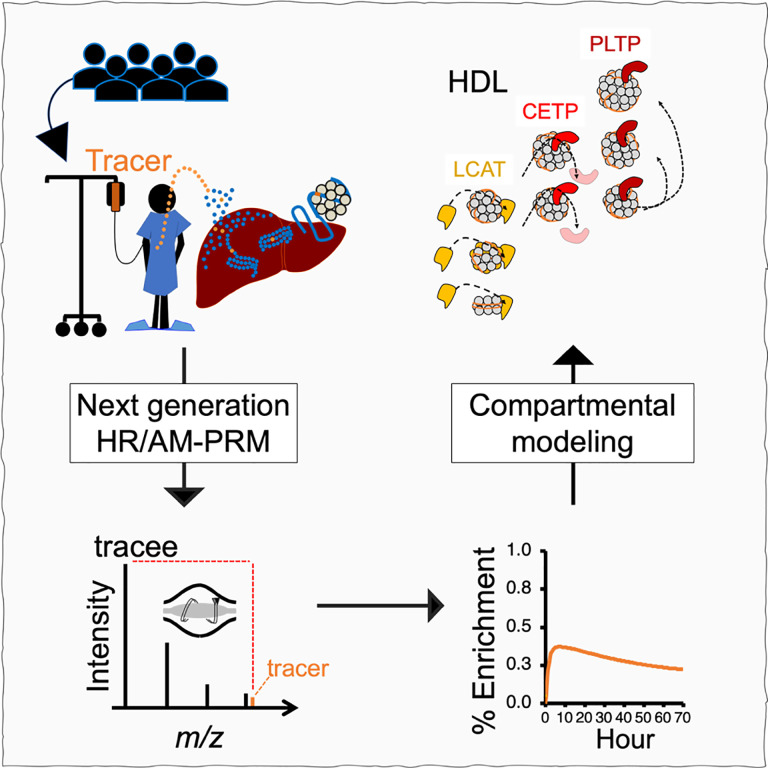

## Introduction

The metabolic properties of circulating apolipoproteins have been intensely studied (1–3), especially since elevated levels of LDL-cholesterol and VLDL-triglycerides, and low levels of HDL-cholesterol, are associated with cardiovascular risk (4–6). Statin therapies have been largely successful at lowering LDL-cholesterol and cardiovascular risk. On the other hand, HDL-cholesterol–raising trials have yielded confounding results that outweigh potential benefits to decreasing coronary events (7–9).

Recent studies into the metabolism of HDL proteins in humans have employed endogenous labeling with stable isotope tracers, such as trideuterated leucine (D3-Leu) or dideuterated water (1, 10–12). Tracer detection in peptides has relied primarily on unit resolution triple-quadrupole platforms to perform multiple reaction monitoring (MRM) (1). However, the reliance on unit resolution readouts to measure low abundant 2HM3 deuterium tracer has limited most studies to total HDL protein pools, such as total apolipoprotein A1 (APOA1), the major structural protein of HDL. The majority of HDL proteins are low abundant (>100-fold less than APOA1) and are slowly metabolized, making tracer detection challenging for low-resolution instruments (1). In 2016 we demonstrated that parallel reaction monitoring (PRM) performed on the high resolution/accuracy mass (HR/AM) quadrupole Orbitrap (Q Exactive) could differentiate D3-Leu’s 2HM3 ion from its natural M3 isotopolog and baseline ions for multiple HDL proteins, in up to 5 HDL sizes (13). Using the PRM approach, Andraski et al. demonstrated that dietary complex carbohydrates, when replacing dietary unsaturated fat, induced a hypermetabolic state of several HDL proteins on specific HDL sizes (14). Due to the ability for HR/AM technology to detect low abundant tracer signals, the HDL protein-specific responses to a diet intervention were reported for the first time.

HDL is a complex population of lipoproteins that vary in lipid and protein content as well as in particle size and shape (14–16). HDL particle heterogeneity is in part regulated by the lipid transfer proteins lecithin-cholesterol acyl transferase (LCAT), phospholipid transfer protein (PLTP), and cholesterol ester transfer protein (CETP). LCAT regulates the size, shape, and lipid composition of HDL particles by esterifying free cholesterol in very small discoidal prebeta and small spherical alpha3 particles, converting them to larger, spherical alpha2 and alpha1 HDL (17, 18). PLTP also regulates the lipid composition and increases the size of HDL particles by transferring phospholipid from APOB-lipoproteins to HDL (19), and by mediating phospholipid transfer between HDL particles, leading to HDL particle fusion and size expansion (20, 21). CETP regulates HDL lipid composition by exchanging cholesterol ester in large HDL with triglycerides from apolipoprotein B– lipoproteins (APOB-lipoproteins) (22). Given their critical roles in lipoprotein metabolism, these enzymes are valued as potential targets for the prevention of coronary heart disease (23, 24). To date, however, only inhibition of CETP and activation of LCAT (via infusion of recombinant proteins) have made it to intervention trials. CETP inhibitor trials aimed to reduce coronary events by promoting preferential flux of cholesterol esters away from proatherogenic APOB-lipoproteins and toward the HDL clearance pathway (24). Despite increases in HDL-cholesterol, CETP inhibitor trials have been complicated by the paradoxical small to no reduction in coronary events and by other unexpected outcomes, leading one to question the future of CETP drugs (9, 25). Early-phase LCAT therapy trials have been completed or are underway (26). Infusion of recombinant LCAT is expected to promote cholesterol esterification and clearance through the HDL clearance pathway (26). In addition, PLTP, CETP, and LCAT’s plasma concentrations or activities have been included as outcome measures in other intervention trials (e.g., ClinicalTrials.gov identifiers NCT03948295, NCT00240305), further underscoring their recognized value to elucidate mechanisms that can correct dyslipidemia.

Despite their critical roles in regulating HDL particle lipid composition, and pursuits to study these enzymes in a clinical setting, the evidence for the in vivo metabolism of PLTP, CETP, and LCAT on HDL in humans remains scant. However, recent studies of HDL metabolism in humans, some of which utilized PRM, determined the metabolism of APOA1, as well as several additional HDL proteins 100s-fold less abundant than APOA1, on multiple HDL sizes (13, 14, 27). Compartmental modeling in these studies showed that size expansion pathways representing conversion of small to larger HDL comprises only about 10%–12% of total APOA1 flux (13, 14). Instead, the majority of APOA1 on each alpha HDL size is secreted into circulation and in turn is cleared from that size (13, 14, 27). A similar metabolic structure was seen for the other HDL proteins monitored (13, 14). These findings suggest that size expansion is not a universal characteristic of HDL particles but instead may only occur within specific protein or lipid-containing HDL subspecies. We speculate that these size expansion subspecies may interact with or contain PLTP, CETP, and/or LCAT. Despite efforts in our laboratory to monitor PLTP, CETP, and LCAT metabolism in these recent studies, their low abundances in plasma (>1000-fold less than APOA1 in plasma) made it difficult to measure tracer consistently across individuals in multiple HDL sizes, even using the Q Exactive. To date, only the metabolism of immunoprecipitated CETP from plasma has been reported (28) but not the metabolism of CETP on specific HDL sizes. Additionally, LCAT metabolism on alpha3 HDL, where most LCAT resides (14, 29), was recently reported, but the metabolism of LCAT on additional HDL sizes remains undetermined. Finally, there are no reports on the metabolic properties, in humans, of PLTP.

In this study, we evaluated the ability for a more recent HR/AM platform, the Orbitrap Lumos (released in 2015, compared to the Q Exactive released in 2011), to detect tracer enrichment in PLTP, CETP, and LCAT in multiple HDL size fractions. We predicted that the Lumos, with its higher resolving power, higher signal-to-noise, lower limit of detection, and more advanced quadrupole, relative to the Q Exactive, would be able to accurately and consistently detect tracer in these low abundant proteins across multiple HDL sizes. To benchmark our methods, we first compared APOA1 and apolipoprotein E (APOE) enrichment data collected on both the Q Exactive and Lumos, primarily to identify sources of tracer enrichment variation that are more readily mitigated by the Lumos. We chose to monitor APOA1 and APOE for these interinstrument comparisons, as the metabolism of these proteins across the HDL sizes are well established (13, 14, 30). Subsequent enrichment analysis performed on the Lumos of HDL isolated from 6 participants showed that the metabolism of PLTP and CETP was associated with the middle to large HDL sizes (alpha2, alpha1, and alpha0) and LCAT associated with small to middle HDL sizes (prebeta, alpha3, and alpha2). Our findings demonstrate that each lipid transfer protein exhibited a unique metabolic structure, likely reflecting its distinct function(s) in vivo. Moreover, we provide technical considerations for future studies aiming to perform metabolic labeling of these or other low abundant proteins.

## Results

### PRM-enabled clinical metabolism studies.

**Figure 1:**
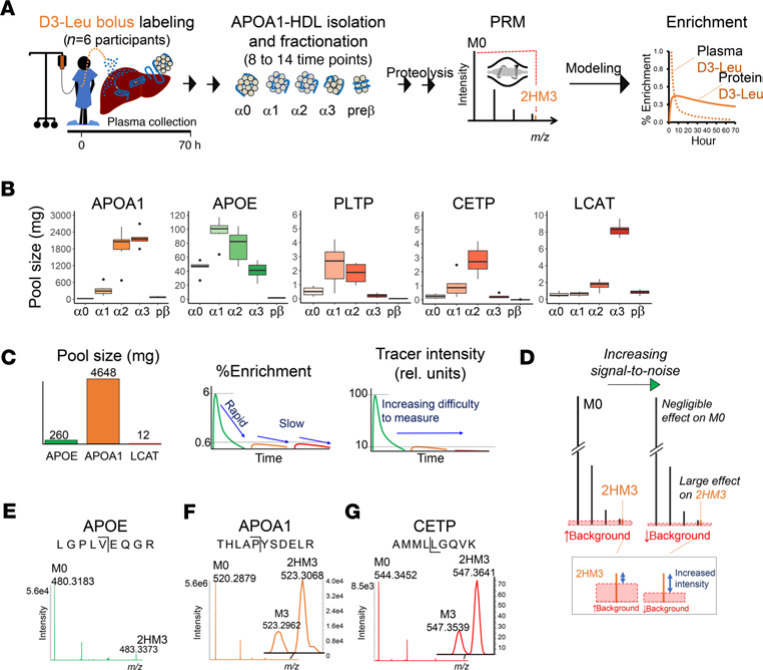
D3-Leu tracer detection in HDL proteins by PRM. (**A**) Administration and detection of D3-Leu in HDL proteins. A 10-minute intravenous bolus injection of D3-Leu at 10 mg/kg per participant is the formulation for the bolus dose. Plasma HDL was isolated and fractionated into sizes using native gel electrophoresis. PRM is required to detect D3-Leu incorporation into HDL proteins. Kinetic parameters were determined by compartmental modeling. (**B**) Protein pool sizes per HDL size fractions calculated using stable isotope peptide standards and ELISA, for subsequent compartmental modeling (data points, *n* = 6 participants). (**C**) Total HDL protein pool sizes and enrichment curve schematics of their relative rapid versus slow turnover rates and the consequence for tracer detection. (**D**) Increasing signal-to-noise improves tracer (2HM3) detection with negligible effect on the tracee (M0). (**E**–**G**) Example MS2 isotope clusters that highlight the range in absolute signals between M0 and tracer 2HM3 peaks within and between peptide fragments. The time point is 4 hours postbolus. Resolution (R) = 240 K at *m/z* 200 on the Lumos. Intensity, normalized level counts.

Our laboratories developed a PRM-based approach to monitor tracer enrichment in plasma HDL proteins to determine their metabolic properties (Figure 1A and refs. 13, 14, 29). In our program, participants are administered an intravenous bolus injection of D3-Leu that is taken up by the liver and other tissues and incorporated into newly translated proteins. We specifically study proteins that circulate on 1 or more APOA1-HDL particle sizes (alpha0, alpha1, alpha2, alpha3, and prebeta). As the resolution of the PRM scan increases, the ability to detect the D3-Leu tracer (2HM3 ion) increases, permitting reliable identification of the tracer that can be 100s-fold to 1000s-fold less abundant than that of D0-Leu tracee (M0 ion) peak (29). The resulting enrichment curves are used as input data into compartmental modeling software (SAAM II) to calculate kinetic parameters such as the fractional catabolic rate (FCR) and production rate of each protein per HDL size (Figure 1A). Studies using the Q Exactive demonstrated that bolus-administered tracer does not surpasses 1% peak enrichment in most HDL proteins studied to date (13, 14, 29). These studies, however, were limited to proteins whose abundances are 100s-fold less abundant than APOA1. In the following sections, we demonstrate the benefits of transitioning to a more advanced MS platform, the Orbitrap Lumos, to determine tracer enrichment in proteins whose abundances are 1000s-fold less than that of APOA1, specifically, PLTP, CETP and LCAT.

### Tracer detection is challenging for low abundant, slowly metabolized proteins.

To underscore the challenges associated with in vivo tracer enrichment studies, we first provide an overview of the reliance on protein pool sizes and turnover rates on the ability to detect tracer. For instance, the total APOE pool size is approximately 20-fold lower than that of total APOA1 (Figure 1, B and C; Supplemental Figure 1; and Supplemental Tables 1 and 2; supplemental material available online with this article; https://doi.org/10.1172/jci.insight.143526DS1), yet due to its rapid metabolism relative to APOA1, APOE’s peak enrichment is approximately 10-fold higher (~7% compared with ~0.6% for APOA1; Figure 1C; and refs. 13, 14). As a consequence, APOE’s tracer can be measured in the MS1 scan that has high interference, although with higher variability than with PRM (MS2 scan, ref. 13). On the other hand, the ability to detect APOA1’s low tracer is compensated by its abundant pool sizes (Figure 1C, Supplemental Figure 1, and Supplemental Table 1) and corresponding intense MS signal. Nonetheless, APOA1 enrichment must be detected in the MS2 scan because the high interference in MS1 results in significant enrichment curve compression that can thus lead to inaccurate kinetic parameter calculations (13, 31). Curve compression occurs when the majority of the tracer (2HM3) peak intensity falls below background signal, resulting in a lower-than-expected peak measurement; whereas the tracee (M0) peak is high enough in intensity that signal loss owing to background effects is negligible (Figure 1D). Proteins, such as PLTP, CETP and LCAT, whose total HDL pool sizes are even lower than that of APOE (Figure 1C) but that are slowly metabolized like APOA1, are more vulnerable to curve compression and thus the most challenging to study. For instance, looking at the Lumos-generated PRM scans from HDL alpha2 (4 hours postbolus), CETP’s tracee (M0) intensity (8.5e3 counts) is approximately 7-fold less than that of APOE’s tracee peak (5.6e4 counts) and considerably less than that of APOA1’s tracer (2HM3) peak (4.0e4 counts). CETP’s tracer intensity is lower yet, at 73 (Figure 1, E–G). Despite the large dynamic ranges in peak intensities, these examples demonstrate that tracer is in theory detectable. To determine the metabolic parameters of a protein, however, tracer detection must be reliable across the study period and across participants. A major source of technical variance that can compromise reliability is tracer enrichment compression (29, 32), to which low MS signals are particularly vulnerable. In the following section, we demonstrate the ability for the Lumos to mitigate sources of enrichment compression.

### The Lumos improves detection of low tracer ions and alleviates enrichment compression.

Before pursuing a full HDL enzyme metabolic profile for PLTP, CETP, and LCAT using the Lumos, we ran interinstrument comparisons using APOA1 and APOE. We evaluated the impact of sample injection dilution and isolation window (varying the isolation mass range around precursor M0 and 2HM3 peaks for coisolation) on enrichment variance. Sample injection must be considered carefully since overfilling the Orbitrap can result in peak coalescence (33) that in turn would affect enrichment calculations. We used the 0.5-hour time point because, as the lowest 2HM3 signal, it is most vulnerable to measurement error due to interference. We used HDL sizes alpha1 and alpha3 since their respective protein pool sizes for APOA1 and APOE vary in intensity, providing us a biologically relevant dynamic range to test. We determined that neither sample dilution nor isolation window influenced the 0.5-hour enrichment data as much as the instrument platform itself (Supplemental Figure 2 and Supplemental Table 3). Specifically, APOA1 0.5-hour enrichment was slightly higher on the Lumos (alpha1, 0.15%; alpha3, 0.07%) than on the Q Exactive (alpha1, 0.1%; alpha3, 0.04%) across the majority of injection dilution and isolation windows (Supplemental Figure 2), likely reflecting the improved signal-to-noise capability of the Lumos (34). In contrast, APOE 0.5-hour enrichment was similar between the 2 platforms (alpha1, 1.5%; alpha3, 2%; Supplemental Figure 2), which was also expected, owing to APOE’s rapid metabolism and >10-fold higher tracer peak signal (relative to APOA1) that is less vulnerable to noise (Supplemental Figure 2). Given that the APOA1 0.5-hour enrichment was slightly higher on the Lumos compared with the Q Exactive, we investigated whether this alleviation of enrichment compression was unique to the early time point or representative of all time points in the experiment. In the following section we analyzed the entirety of the time course data for APOA1 and APOE to determine to what extent, if any, enrichment compression alleviation may affect compartmental modeling.

### APOA1 and APOE metabolic rates are similar for the Q Exactive– and Lumos-derived enrichment curve data.

Using similar injection dilutions, but tailoring acquisitions optimal for each instrument platform (see Methods), we collected the enrichment profiles for APOA1 and APOE across the 5 HDL sizes for participant 1. In line with the 0.5-hour APOA1 pilot data above, variance component analysis of APOA1 enrichment across the 5 HDL sizes showed that the lowest intensity tracer ions were vulnerable to enrichment compression and that this vulnerability was more pronounced on the Q Exactive (Supplemental Figure 3, A and B). We also demonstrated that APOA1 enrichment variance for the multiple fragment ions in 14 time points across the HDL sizes was consistently lower on the Lumos than the Q Exactive (Supplemental Figure 3C).

We next used the APOA1 and APOE enrichment data (median of the fragment ions) generated on the Q Exactive and Lumos (Supplemental Figure 4A), and their pool sizes (collected on the Lumos; Supplemental Table 1, participant 1), to calculate and compare metabolic parameters. Metabolic rates, FCR, and production rate of APOA1 and APOE on each HDL size were estimated using our previously established APOA1 and APOE compartmental models (13, 14). Each model contains an input, source, 4 (APOE) or 5 (APOA1) HDL size compartments, and pathways from the source into each HDL size (see Methods, *Compartmental modeling*; Supplemental Figure 4B). The APOA1 model also contains an extravascular delay compartment, a lipidated APOA1 compartment, as well as transfer pathways from alpha3 to alpha2 and prebeta and from prebeta to alpha2, alpha1, and alpha0 (Supplemental Figure 4B). The APOE model contains transfer pathways from alpha3 to alpha0, alpha1, and alpha2 and from alpha2 to alpha0 and alpha1 (Supplemental Figure 4B). The enrichment and pool size data for each protein on each HDL size were uploaded to each HDL size compartment.

The same transfer pathways were detected using the Q Exactive and Lumos enrichment curves for each model: APOA1 transfer from prebeta to alpha0 and alpha1 and from alpha3 to prebeta and APOE transfer from alpha3 to alpha2 were detected (Supplemental Figure 4B). The APOA1 and APOE FCR and production rates, and their trends across the HDL sizes, were also similar between the Q Exactive– and Lumos-generated enrichment curves (Supplemental Figure 4, C and D). The FCR of APOA1 was highest in alpha0 and prebeta, followed by alpha1, and then alpha2 and alpha3. The production rate of APOA1 was highest in alpha2, similar in alpha1 and alpha3, and lowest in prebeta and alpha0 (Supplemental Figure 4C). The APOE FCR and production rate in alpha2 were also highest on both platforms (Supplemental Figure 4C). The consistencies in metabolic rates between the platforms are not surprising given that both instruments and their corresponding compartmental modeling fits were able to detect the different enrichment curve shapes across the HDL sizes. For example, the higher and earlier enrichment peaks of APOA1 alpha0 and alpha1 enrichment and the lower and later enrichment peak of APOA1 alpha3 were consistently detected using both the Q Exactive and Lumos (Supplemental Figure 4A).

The only notable difference between the compartmental modeling results from the 2 instrument platforms was that the spread of the enrichment data points around the model fit tended to be smaller on the Lumos. We quantified the data point spread around the model fit by taking the sum of squared residuals (SSR) for each protein across all HDL sizes and time points for the Q Exactive and the Lumos data. The SSR for APOA1 was 0.39 and 0.30 and for APOE was 23.2 and 8.5 for the Q Exactive and Lumos data, respectively (Supplemental Figure 4E).

### The Lumos alleviates enrichment compression for PLTP and CETP.

We next compared PLTP alpha1 and CETP alpha2 enrichment data acquired on the Q Exactive and Lumos. We did not perform this interinstrument enrichment comparison for LCAT, as we previously reported the Q Exactive–generated enrichment and metabolism of LCAT in alpha3 HDL, the size fraction in which the enzyme predominates (14, 29). PLTP and CETP mainly reside in alpha1 and alpha2 HDL with PLTP more so in alpha1 and CETP in alpha2 (Figure 1B and ref. 14). Their relative absolute quantities are 100s-fold (alpha1) to 1000s-fold (alpha2) less than that of APOA1, as determined by stable isotope dilution quantification (Supplemental Figure 1).

**Figure 2:**
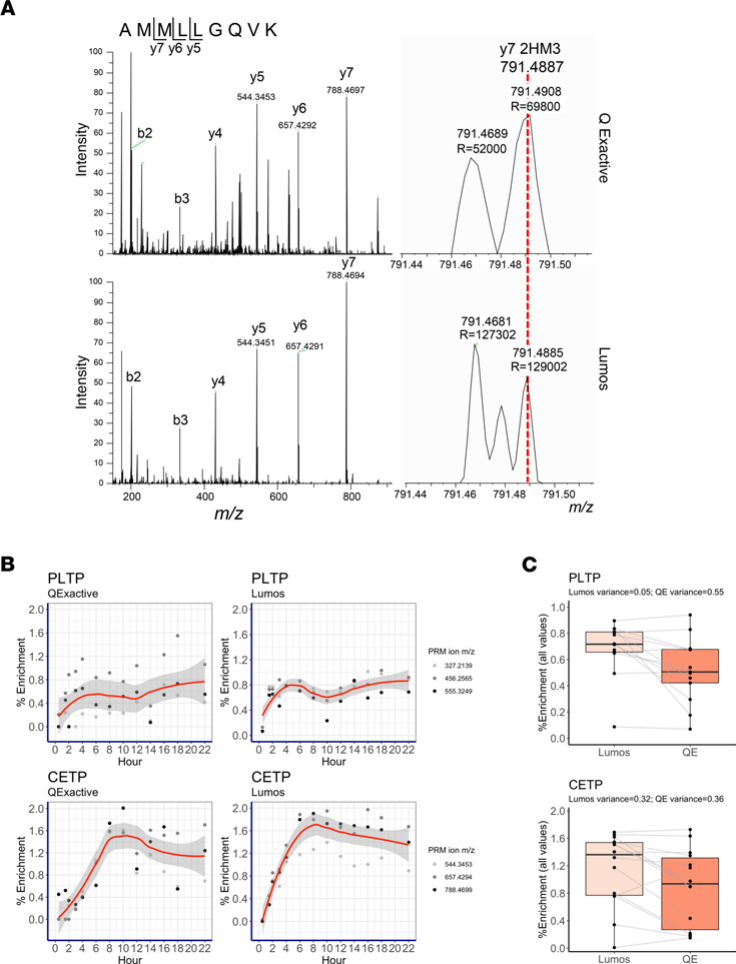
An interinstrument platform comparison of PLTP and CETP tracer enrichment data. (**A**) PRM scans of the same CETP peptide from the Q Exactive and the Lumos. The relative peak intensities of the fragment ions are conserved between the 2 instruments. The *y7* 2HM3 (tracer) peak environment is zoomed in. R = 120 K for the Q Exactive and 240 K for the Lumos. (**B**) Loess regression plots showing that the standard error (gray) of the fitted curves is lower on the Lumos. PLTP data are from alpha1 and CETP from alpha2. Legend: the PRM ions’ *m/z* values. (**C**) Box plots depicting the distribution of the enrichment data in **B**. Each data point is the average enrichment (*n* = 3 PRM ions’ measurements) per time point. The box plots depict the minimum and maximum values (whiskers), the upper and lower quartiles, and the median. The length of the box represents the interquartile range. The gray lines indicate the relative shift in enrichment value per given time point. Variance was calculated using individual PRM ion enrichment data in **B**, not the averages.

To further illustrate the benefits of using the Lumos for tracer detection in these low abundant and slowly metabolized proteins, we compared enrichment data collected on the Lumos and Q Exactive for PLTP and CETP. Using samples from participant 2, we diluted alpha1 and alpha2 peptide stocks to an estimated 114 attomoles of PLTP (alpha1) and 128 attomoles of CETP (alpha2) on column. It is important to note that the peptides used for absolute quantification of PLTP and CETP were distinct from those that were used for enrichment because the latter did not meet criteria for absolute quantification (see Methods, *Absolute quantification of peptides*). We monitored enrichment in the same 3 Leu fragments for each enzyme, including the *y6* and *y7* ions for CETP (*m/z* 657.4292 and *m/z* 788.4697, Figure 2A) that fall outside of the recommended mass range (*m/z* < 600) for enrichment analysis on the Q Exactive (13). Both instruments captured the unique enrichment curve representing each enzyme. For instance, both Lumos and Q Exactive enrichment curves depicted 2 peaks for PLTP in alpha1; the first at 6 hours and the second at 20 hours; however, the peaks’ enrichments were slightly higher in the Lumos curves — 0.8% for the Lumos versus 0.6%–0.7% for the Q Exactive (Figure 2B).

For CETP in alpha2, a single enrichment peak defined this enzyme; however, its enrichment of 1.6% spanned 8 to 12 hours in the Q Exactive curve, whereas it was slightly higher (1.8%) and distinct at 8 hours for the Lumos curve (Figure 2B). While the general curve shapes were the same between the instruments, overall enrichment was higher on the Lumos (Figure 2C). Enrichment data variance was comparable between the 2 instruments for CETP but lower on the Lumos for PLTP (Figure 2C). A closer look at CETP’s enrichment data shows that while variance was similar between the 2 instruments, the cause of the variance was distinct (Figure 2B). On the Q Exactive, all 3 ions were randomly scattered around the regression curve (Figure 2B); on the other hand, on the Lumos, each ion exhibited a similar curve shape, but the *y5* curve (*m/z* 544.3453) was lower than those of the *y6* (*m/z* 657.4294) and *y7* (*m/z* 788.5699) ions.

### PLTP, but not CETP, transfer occurs among the large HDL sizes.

**Figure 3:**
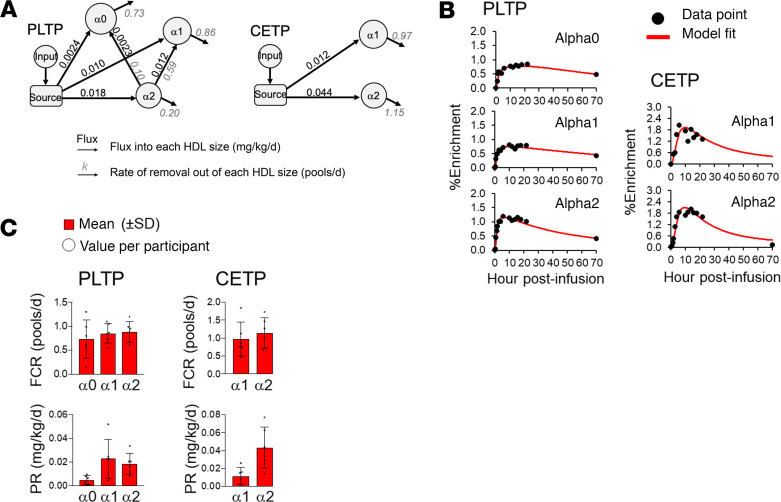
Compartmental models and kinetics parameters for PLTP and CETP in the larger alpha HDL size fractions. (**A**) Compartmental model for PLTP and CETP (the average of *n* = 6 participants). PLTP flux into alpha1 is approximately 45% from the source (0.010 mg/kg/d) and 55% from the smaller alpha2 (0.012 mg/kg/d). Approximately 75% of PLTP on alpha2 is transferred to alpha0 and alpha1 (0.10 and 0.59 pool/d, respectively) while the remaining 25% is removed from the model system (0.20 pool/d). CETP appears on alpha1 and alpha2 via direct secretion. (**B**) Enrichment curve fits generated from the models in **A**, participant 1. (**C**) FCR and production rate (PR) for PLTP and CETP. Bar graphs represent the mean value for *n* = 6 participants, error bars represent SD, and open circles represent values per participant.

We next used the Lumos-generated PLTP and CETP enrichment values from 6 participants to create compartmental models that describe the metabolism of each enzyme on alpha1 and alpha2 HDL. Because PLTP tracer was detected in alpha0 for all participants, we also modeled PLTP in alpha0. PLTP enrichment curves differed across the HDL sizes: PLTP on alpha2 showed a single enrichment peak between 2 and 12 hours (0.9%–1.6% enrichment), while PLTP on alpha0 and alpha1 showed 2 enrichment peaks, the first between 1.5 and 12 hours (0.4%–0.9% enrichment) and the second between 12 and 22 hours (0.4%–1.1% enrichment, Supplemental Figure 5A). The appearance of the second peak in alpha0 and alpha1, which appeared later than the single peak in alpha2, suggested that PLTP on alpha2 may transfer to the larger alpha1 and alpha0 HDL sizes. The PLTP model contained an input and source compartment; 3 HDL size compartments representing PLTP on alpha0, alpha1, and alpha2 HDL; direct secretion pathways from the source into each HDL size; and transfer pathways from alpha2 to alpha0 and alpha1 (Figure 3, A and B). On the other hand, CETP enrichment curves tended to look similar between the alpha1 and alpha2 HDL sizes, with no indication of transfer pathways. For instance, the enrichment curves for both sizes peaked between 4 and 12 hours with peak enrichments of 1% to 3% (Supplemental Figure 5A). Additionally, the CETP enrichment curves were highly variable across the 6 participants (Supplemental Figure 5A). The CETP model thus contained an input and source compartment (Methods, *Compartmental modeling*), 2 HDL size compartments representing CETP on alpha1 and alpha2 HDL, and direct secretion pathways from the source into each HDL size (Figure 3, A and B).

### Over half of PLTP flux into alpha1 originates from alpha2, while all CETP flux originates from the source.

The PLTP alpha2 to alpha0 transfer pathway was detected in 3 participants and from alpha2 to alpha1 in all 6 participants (Supplemental Figure 5B). Sixty-nine percent of total PLTP flux into alpha0 originated from the source, while 31% originated from alpha2; 45% of total PLTP flux into alpha1 originated from the source, with 55% from alpha2 (Supplemental Figure 5B). On average, the majority of PLTP on alpha2 was transferred to alpha1 (64%), with smaller amounts transferred to alpha0 (11%) and removed out of the model system (25%, Supplemental Figure 5C). The average PLTP FCR on alpha0, alpha1, and alpha2, was 0.73, 0.85, and 0.88 pool/d, respectively (Figure 3C and Supplemental Table 4), and the average production rate was 0.0047, 0.023, and 0.018 mg/kg/d, respectively (Figure 3C and Supplemental Table 4). In contrast, all CETP flux into alpha1 and alpha2 HDL originated from the source and was subsequently removed from that same size; CETP transfer from alpha1 to alpha2 and from alpha2 to alpha1 was not detected (Figure 3A). The average CETP FCR on alpha1 and alpha2 was 0.97 and 1.15 pools/d, respectively, and the average production rate was 0.012 and 0.044 mg/kg/d, respectively (Figure 3C and Supplemental Table 4).

### LCAT appearance in circulation is delayed on the small HDL sizes.

Most HDL-associated LCAT is found in alpha3, but small amounts are detectable in all sizes, most notably, alpha2 and prebeta (Figure 1B and refs. 13, 14). Potentially unique to this study, using the Lumos, tracer was detected not only in alpha3 but also in alpha2 and prebeta in all 6 participants (Supplemental Figure 6). Interestingly, the appearance of LCAT tracer in each HDL size was delayed and did not appear in circulation until 1 to 6 hours postinfusion (Supplemental Figure 6), unlike PLTP and CETP, which showed more rapid appearance, by 30 minutes and 1 hour, respectively (Supplemental Figure 5A). Additionally, in 4 of 6 participants, LCAT on prebeta tended to appear in circulation between 3 and 6 hours, later than LCAT on alpha3 at 1 to 4 hours. The overall LCAT enrichment curve shapes tended to look similar across the HDL sizes: the enrichment curves for all sizes peaked between 6 and 22 hours with peak enrichment of 0.30% to 0.86% (Supplemental Figure 6).

**Figure 4:**
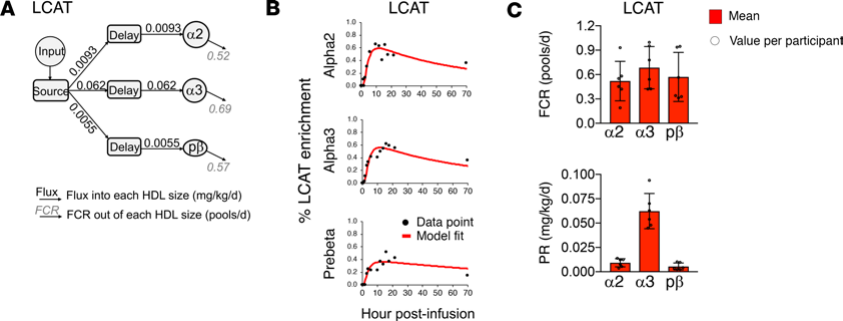
Compartmental model and kinetics parameters for LCAT in the smaller alpha and prebeta HDL. (**A**) Compartmental model for LCAT (the average of *n* = 6 participants). (**B**) Enrichment curve fits generated from the model in **A**, participant 1. (**C**) FCR and PR for LCAT. Bar graphs represent the mean value for *n* = 6 participants, error bars represent mean ± SD, and open circles represent values per participant.

The resulting LCAT model contained an input and source; 3 delay compartments that accounted for the delayed appearance of LCAT on each HDL size; 3 HDL size compartments representing LCAT on alpha2, alpha3, and prebeta; and direct secretion of LCAT on each size from the source, through the delay, and into each HDL size (Figure 4, A and B). Transfer pathways among the size fractions were tested. Prebeta to alpha2 and alpha3 to prebeta transfers were detected but only in a single participant for each transfer pathway (participants 4 and 3, respectively). Since these transfer pathways were not detected in 2 or more participants, they did not meet our criteria for being included in the final LCAT kinetic model (Methods, *Compartmental modeling*, Figure 4A). All LCAT flux into alpha2, alpha3, and prebeta originated from the source via a delay and was subsequently removed from that size (Figure 4A). The average FCR of LCAT on alpha2, alpha3, and prebeta was 0.52, 0.69, and 0.57 pool/d, respectively, and the average production rate was 0.0093, 0.062, and 0.0055 mg/kg/d, respectively (Figure 4C and Supplemental Table 4).

## Discussion

In this study, we leveraged recent advances in HR/AM–mass spectrometry to detect tracer enrichment in multiple HDL sizes of low abundant, slowly metabolized enzymes: PLTP, CETP, and LCAT. Each enzyme exhibits unique metabolic properties — PLTP transfers from alpha2 HDL to larger alpha1 and alpha0 HDL; CETP associates with primarily alpha1 and alpha2 and stays on these particles until it is cleared; and LCAT mainly resides in alpha2, alpha3, and prebeta, but its appearance in these sizes is markedly delayed, when compared with PLTP and CETP. These findings are consistent with accumulating evidence that proteins on HDL have unique metabolic properties that may in part modulate HDL function (35).

To date, HR/AM PRM has determined the metabolism of 10 HDL proteins (13, 14). This current study underscores the continued reliance on developing mass spectrometry technologies to conduct stable-isotope tracer studies. We previously demonstrated the advantages of HR/AM PRM on the Q Exactive quadrupole Orbitrap over unit resolution MRM on triple quadrupoles for detecting HDL proteins 100s-fold less abundant than APOA1 (1, 13). In this study, however, we emphasize that continuing developments in HR/AM–mass spectrometry itself, as provided by the Orbitrap Fusion Lumos, increase further the capability to detect tracer enrichment in proteins, such as PLTP, CETP, and LCAT, that are 1000s-fold less abundant than APOA1.

Both Q Exactive and Lumos perform PRM with a similar HR/AM dynamic range of 5000. The Lumos, however, is a recent generation instrument with a higher resolving power, a higher signal-to-noise, a lower limit of detection, and an advanced quadrupole that is more efficient and uniform at coisolating tracer and tracee peaks (36). A higher resolving power can increase the *m/z* limit for candidate fragment ions to measure tracer; a higher signal-to-noise reduces ratio compression by lowering background interference; and improved isolation efficiency can allow for smaller isolation windows around the target tracer and tracee peaks, also reducing interference. Although the Q Exactive could detect tracer in PLTP, CETP, and LCAT, those measurements were limited to only the HDL size in which most of each enzyme resides (alpha1, alpha2, and alpha3, respectively). Q Exactive enrichment data on PLTP or CETP were more variable and slightly compressed when compared with data acquired by the Lumos (Figure 2). Similarly, APOA1 enrichment variance decreased on the Lumos compared with the Q Exactive, but the relative differences in enrichment curves and resulting model fits were conserved between the 2 instruments’ data (Supplemental Figure 4). This conservation is not unexpected since in previous studies using the Q Exactive tracer detection methods had been carefully optimized (13, 29). On the other hand, the increase in sensitivity and precision in mass spectrometric measurements that are required to study, for example, PLTP, CETP, and LCAT metabolism, is why next-generation quadrupole HR/AM instruments, such as the Lumos, are continually being designed (36). Moreover, HR/AM PRM can also be performed on ever-evolving quadrupole time-of-flight mass spectrometers (37), which provide similar advantages to the quadrupole Orbitrap over low-resolution triple quadrupoles (38). Future in vivo metabolism studies should therefore leverage the availability of the combined build of a quadrupole filter with an HR/AM mass analyzer to perform similar in vivo metabolism research.

Leveraging the ability of the Lumos to expand tracer detection to relatively low protein pool sizes, we were able to monitor the metabolism of PLTP in multiple HDL sizes. PLTP alters the size of HDL particles by mediating particle fusion, as shown in vitro, which in turn leads to the generation of larger alpha HDL (20, 21). We identified an average of 5 mg (2.55, SD) of PLTP on HDL, the majority of which (~50%) was on alpha1, with smaller amounts on alpha2 (~36%) and alpha0 (~10%, Figure 1B and Supplemental Table 1). Interestingly, we found that the majority of PLTP on alpha2 (75%) was transferred to larger sizes, alpha1 and alpha0 HDL (Figure 3 and Supplemental Figure 5). We did not detect PLTP transfer between alpha1 and alpha0, suggesting that the majority of PLTP activity may be confined to alpha2 HDL. This finding is consistent with that of previous reports showing that, despite the lower mass of PLTP in alpha2 relative to alpha1, PLTP’s phospholipid transfer activity is localized to alpha2, while PLTP on larger alpha1 is inactive (30, 39). Thus, the PLTP transfer we detect from alpha2 to alpha1 and alpha0 likely represents HDL particle size expansion from alpha2 to larger alpha1 and alpha0 HDL via PLTP, potentially via particle fusion. It also suggests that PLTP remains on the particle as it expands in size.

If the transfer of PLTP on alpha2 to alpha1 and alpha0 is representative of alpha2 particle fusion and size expansion to form alpha1 and alpha0 particles in vivo, we would also expect to see size expansion of alpha2 to alpha1 and alpha0 in our APOA1 model. However, we only detected APOA1-HDL particle expansion from prebeta to alpha0, alpha1, and alpha2, and from alpha3 to alpha2, but not from alpha2 to alpha1 and alpha0 (Supplemental Figure 4B and refs. 13, 14). This interesting discrepancy may suggest that only a minor population or a subspecies of alpha2 HDL undergoes PLTP-mediated particle fusion and size expansion, and the number of alpha2 HDL particles expanding in size may be too low to detect in the APOA1 tracer data. Based on our pool size data, and the assumption that alpha2 contains 4 molecules of APOA1 (40), only 1 molecule of PLTP is present for every 500 particles of alpha2 HDL. If PLTP binds irreversibly to HDL, only approximately 0.2% alpha2 HDL would increase in size. In addition to isolating APOA1-HDL, enrichment of PLTP-containing HDL particles may be necessary to detect APOA1 transfer from alpha2 to alpha1 and alpha0. It is also possible that PLTP’s primary function on HDL in plasma is not its phospholipid transfer activity, which is localized to its minor alpha2 size (30, 39), but instead a currently unidentified function in alpha0 and alpha1 (41). Alpha0 and alpha1 contain the majority of PLTP mass on HDL (60%) and a higher ratio of PLTP per particle (1 molecule of PLTP for every 20 and 50 particles of alpha0 and alpha1, respectively), compared with alpha2 (36% of total PLTP mass on HDL, 1 molecule of PLTP for every 500 alpha2 particles). PLTP on these large HDLs, although inactive in phospholipid transfer, may instead play a role in the immune response: PLTP binds to lipopolysaccharide on gram-negative bacteria, neutralizing its inflammatory effects (42); PLTP deficiency increases mortality after lipopolysaccharide injection in mice (43); and other proteins known to play a role in immunity (complement factors, immunoglobulins, and apoL1) are enriched in alpha0 and alpha1 (14).

Similar to PLTP, CETP was enriched in the large HDL sizes, 20% in alpha1 and 70% in alpha2 HDL, and 1 molecule of CETP was present for every 100 alpha1 and for every 250 alpha2 particles (Figure 1). Since CETP’s primary function is to exchange cholesterol ester in HDL for triglyceride in APOB-lipoproteins, it is not surprising that CETP dominates in large HDL, as these large particles have higher amounts of cholesterol ester compared with small HDL (16). The cholesterol ester in these large particles is likely derived from the liver during HDL particle synthesis, as suggested by the direct secretion pathways into alpha1 and alpha2 HDL in our APOA1 model (Supplemental Figure 4B), and from the esterification of free cholesterol by LCAT, which converts small prebeta and alpha3 to larger CETP-residing alpha2 and alpha1 (17, 18, 44) (see LCAT paragraph below for further LCAT discussion). Unlike PLTP, we did not detect CETP transfer between alpha1 and alpha2. Instead, all CETP on alpha1 and alpha2 originated directly from the source compartment, likely representative of the liver; followed by removal from each of these sizes out of the model system (Figure 3). These findings suggest that CETP-mediated exchange of cholesterol ester for triglyceride may not alter the size of the HDL particle in humans in vivo. These findings are consistent with in vitro data showing that CETP-mediated cholesterol ester and triglyceride transfer between spheroidal reconstituted HDL and VLDL does not change HDL size (45). However, other in vitro studies using reconstituted HDL and intralipid have shown that CETP reduces the size of the HDL particle (46), while studies using ultracentrifugation-isolated human HDL have shown that CETP converts HDL3 (8.7 nm particle diameter) to larger (9–10 nm) and smaller (7.8 nm) HDL particles (47). The discrepancy in findings across studies may be due to varying protein and lipid compositions across the HDL particles. The reconstituted HDL in some of these studies only contained APOA1, while the HDL that binds CETP in vivo likely contained additional proteins. Moreover, ultracentrifugation-isolated human HDL contained less than half the number of proteins compared with HDL isolated by other methods, such as APOA1-immunoaffinity purification used in our study (48). We speculate that the additional proteins on CETP-containing HDL in vivo may regulate the amount of lipid that can enter and exit the HDL core, maintaining its size. Isolating CETP-containing HDL particles by immunoaffinity purification or other ultracentrifugation-independent methods (49) may therefore provide candidate proteins that regulate lipid transfer and HDL size in humans in vivo. The proposed mechanism(s) of CETP-mediated lipid exchange between lipoproteins also vary. Structural studies provide evidence for ternary (HDL-CETP-LDL/VLDL, refs. 50, 51) and nonternary (52) lipid transfer mechanisms. Despite the different findings of each study, they both confirm a stable HDL-CETP complex that is mediated by burial of CETP’s N-terminal tip into HDL. Ongoing or future dyslipidemia trials involving CETP inhibition would therefore benefit from studying its HDL-bound metabolic properties. Altogether, these findings and ours advocate for the isolation and metabolic profiling of CETP in complex with HDL, when trying to understand the mechanisms of action of ongoing or future CETP inhibitors.

LCAT is primarily localized to smaller HDL, most notably the alpha3 size (Figure 1). Since LCAT esterifies free cholesterol and alters the shape (discoidal to spherical) and increases the size of HDL (17, 18, 44), we expected to detect LCAT transfer from small to large HDL. However, despite our efforts to monitor tracer in LCAT on large alpha0 and alpha1, LCAT abundance and turnover were both too low to acquire reliable tracer data in these sizes (Figure 1B and Figure 4). We were able, however, to determine the metabolism of LCAT on prebeta, alpha3, and alpha2 but did not observe an appreciable amount of LCAT transfer between these sizes. Although we did not observe LCAT transfer, this does not mean LCAT is not active in size expansion. It just suggests that LCAT itself may not remain on the HDL particle as it grows in size. In our APOA1 kinetic model, we observe conversion of APOA1 on small prebeta and alpha3 to larger alpha0, alpha1, and alpha2 HDL (Supplemental Figure 4C) and speculate that LCAT activity likely accounts at least partially for these conversions.

Additionally, unlike PLTP and CETP, which appeared on HDL in circulation by 30 minutes and 1 hour, respectively, LCAT appearance on HDL in plasma was markedly delayed and did not occur until 1 to 6 hours postinfusion. The early appearance of proteins, such as PLTP, CETP, and APOA1 and the majority of other HDL proteins, suggests that these proteins may be directly secreted and enter circulation on an HDL particle (13, 14). However, there are several potential mechanisms that may account for the delayed appearance of LCAT on circulating HDL. First, the delayed appearance of LCAT may be due to mechanisms controlling protein synthesis, processing, and secretion from the hepatocyte (its main site of synthesis, ref. 53), that may be slower relative to other HDL proteins. Second, LCAT may be secreted at a similar rate to other HDL proteins, but spends time outside of the systemic circulation, such as in the space of Disse, hepatic sinusoids, interstitial space, or lymphatic vessels, before attaching to circulating HDL (27, 54, 55). It is also possible that LCAT activity in the space of Disse and sinusoids interact with newly synthesized HDL, altering their size and shape before they enter circulation (54, 56). Third, LCAT may be secreted unattached to an HDL particle (57) and enter circulation, where it may interact with other HDL particles, changing their size, before attaching to a circulating HDL particle. In our kinetic modeling system, we only monitor LCAT on circulating HDL. If before attaching to this HDL, LCAT is present in the hepatocyte or an extravascular compartment for an extended period, or in a free form, we cannot directly measure it. However, the presence of these additional LCAT compartments is plausible given the 3 delay compartments unique to the LCAT model. Our findings therefore suggest that future studies into LCAT secretion or metabolism consider additional compartments such as extravascular LCAT and lipoprotein-free LCAT to further delineate the mechanisms underlying LCAT metabolism, function, and contribution of each to HDL heterogeneity.

In summary, our study demonstrates that it is possible to monitor the metabolism of PLTP, CETP, and LCAT in multiple HDL sizes in humans. In addition, we posit that clinical studies addressing particle remodeling or potential protein transfer across HDL subspecies are increasingly feasible with support of evolving HR/AM technologies such as Orbitrap.

## Methods

### Clinical study and samples

We recruited 6 participants with low HDL-C (≤55 mg/dL for females, ≤45 mg/dL for males) and who were overweight or obese (BMI > 25 kg/m^2^) (Supplemental Table 5). Plasma samples from these same participants were also analyzed in our recently published study (14). Exclusion criteria included high LDL-cholesterol (>190 mg/dL); very low HDL-cholesterol (<20 mg/dL); very high fasting triglycerides (>500 mg/dL); *APOE* genotypes E2E2, E2E4, or E4E4; use of medications or therapies that can alter lipid levels; and secondary hyperlipidemia (14). The participants consumed a controlled diet (20% fat [8% monounsaturated, 7% polyunsaturated, 5% saturated], 65% carbohydrate, 15% protein, 90 mg cholesterol) for 4 weeks prior to the kinetics study. The controlled diet adhered to the Institute of Medicine Dietary Reference Intake guidelines for healthy nutrient intake (http://ods.od.nih.gov/Health_Information/Dietary_Reference_Intake.aspx) and was formulated by Brigham and Women’s Hospital Center for Clinical Investigation (CCI) nutrition research unit. All food and beverages were provided for the duration of the study. Alcoholic beverages were not part of the study diet and intake was not permitted. Participants visited the CCI every Monday, Wednesday, and Friday, where they picked up food, completed a food diary, and had their body weight measured. Calories were adjusted to compensate for any complaints of hunger or satiety or changes in body weight.

### Tracer infusion protocol

On the morning of day 28 of the controlled diet, participants were admitted to Brigham and Women’s Hospital CCI, where they received an intravenous bolus injection of the stable isotope tracer D3-Leu at a concentration of 10 mg/kg over 10 minutes. Blood was sampled immediately before the bolus injection (time 0 hour) and at up to 70 hours postinfusion. After the 22-hour sample was collected, participants were discharged. The 46- and 70-hour postinfusion blood samples were collected at the ambulatory CCI. Total plasma leucine (D3-Leu labeled and endogenous) was isolated from 0.2 mL of plasma from time points 0, 1, 2, 3, 4, 6, 8, 10, 12, 14, 16, 18, 22, 46, and 70 hours postinfusion using an AG 50W-X8 cation exchange resin (Bio-Rad). The isolated amino acids were then dried under nitrogen, derivatized to heptafluorobutyric acid esters, and measured using gas chromatography–mass spectrometry (Agilent 6890 GC, 5973 MS). The total plasma tracer (D3-Leu) enrichment was quantified by taking the area under the curve of the tracer divided by the area under the curve of total plasma leucine (D3-Leu tracer + Leu tracee).

### HDL isolation, size fractionation, and proteolysis

HDL sample preparation has been reported in great detail previously (13, 14, 29), but the salient steps are outlined here. For each participant, HDL was isolated from 8 to 14 time points after D3-Leu infusion: the specific time points (listed in Supplemental Table 5) were chosen before data analysis and varied across participants due to sample availability (samples from this same clinical study were analyzed in previous publications, refs. 1, 14, 29). Additionally, our previous work illustrated that the 8 time points chosen for participants 4, 5, and 6 (Supplemental Table 5) were sufficient to detect the unique enrichment curves of APOA1, APOE, LCAT, and several other HDL proteins (1, 14, 29), and we predicted they would be sufficient for PLTP and CETP enrichment as well.

HDL was purified from 1 mL of plasma by overnight incubation with anti-APOA1 immunoglobulin (Academy Biomedical) bound to sepharose 4B resin (Academy Biomedical). The unbound, non–APOA1-containing fraction was collected by gravity flow, and the bound, APOA1-containing fraction was eluted using 3 M NaSCN (MilliporeSigma). Immediately after isolation, APOA1-HDL was separated by size using nondenaturing polyacrylamide gel electrophoresis on a 4%–30% gradient gel (Jule, Inc.) run at 15 mA for 16 hours. A molecular weight standard from the GE/Amersham calibration kit (catalog 17-0445-01) was run alongside the samples. After completion of the run, the gel was stained for 1 to 2 hours in Coomassie Brilliant Blue (Invitrogen, Thermo Fisher Scientific) and destained in double-distilled H_2_O until the gel background was mostly clear. Using the molecular weight standard as a guide, portions of the gel corresponding to each HDL size were excised: above 12.2 nm, alpha0; between 12.2 nm and 9.5 nm, alpha1; between 9.5 nm and 8.2 nm, alpha2; between 8.2 and 7.2 nm, alpha3; and the band at 7.1 nm, prebeta. The excised gel pieces were proteolyzed using trypsin for 4 hours at 37°C using a standard protocol, with the exception that the alkylation step was omitted to increase throughput of sample preparation (13). Peptide samples were resuspended in 5% acetonitrile and 0.5% formic acid dissolved in mass spectrometry–grade water.

### Mass spectrometry

The aim of this study was to understand sources of technical variation and limitation on the acquisition and fidelity of low abundant tracer in vivo, not to match or compare the performance of the Q Exactive with the technically more advanced Lumos platform, per se. Thus, differences in each platform’s constitution (including peripheral devices, such as the column type and temperature) are expected to affect the data quality. However, these differences are minor compared with, for instance, the differences in the ion inlet and optics guiding the eluted peptides into the mass spectrometer (https://planetorbitrap.com, ref. 36). As a consequence, we do not expect that differences in the columns affect the differences in data quality described in this study. Both Q Exactive (quadrupole + Orbitrap) and Orbitrap Fusion Lumos (quadrupole + linear ion trap + Orbitrap) instruments were coupled to an Easy-nLC1000 HPLC pump (Thermo Fisher Scientific). The Lumos was fronted with an EASY-Spray ion source and the Q Exactive with a Nanospray FLEX ion source (Thermo Fisher Scientific).

#### Lumos.

Peptides were separated using a dual-column setup: an Acclaim PepMap RSLC C18 trap column, 75 μm × 20 mm; and a heated EASY-Spray column (45°C), 75 μm × 250 mm (purchased from Thermo Fisher Scientific). The gradient flow rate was 300 nL/min from 8% to 25 % solvent B (acetonitrile/0.1 % formic acid) for 10 minutes, 25% to 95 % solvent B for 2 minutes, followed by an additional 5 minutes of 95 % solvent B. Solvent A was 0.1 % formic acid. Data-dependent acquisitions (DDAs) on the Lumos provided retention times of target HDL proteins. The instrument was set to 120 K resolution, and the top N precursor ions in a 3-second cycle time (within a scan range of 375–1500 *m/z*) were subjected to higher energy dissociation (HCD, collision energy 30%) for peptide sequencing using a 30 K resolution setting. The parallelization feature was enabled (automatic gain control/AGC target, 1.0e5; maximum injection time, 54 ms). PRM was performed using the “targeted MS2 scan” module, in scheduled mode (Supplemental Table 3) when collecting enrichment data for modeling. Dissociation was set to 30% HCD collision energy, and the PRM scans (150–1000 *m/z*) were set to 240 K resolution (AGC target 2.0e5; maximum injection time, 502 ms). PRM data used for modeling were acquired with a 4 Da isolation window on the average of the M0 and 2HM3 (or 2HM6 for peptides with 2 leucines) (Supplemental Table 3).

#### Q exactive.

Peptides were separated using an Acclaim PepMap RSLC C18 trap column, 75 μm × 20 mm; and an Acclaim PepMap RSLC C18 analytical column 75 μm × 250 mm (Thermo Fisher Scientific). PRM was performed using the “DIA” module (these settings can be applied to other tMS2 modules such as “PRM” if available) and in schedule mode, with retention time windows adjusted for the Q Exactive’s chromatography, accordingly. Dissociation was set to 25% HCD collision energy, and the PRM scans (fixed first mass, 100 *m/z*) were set to 140 K resolution (AGC target 1.0e6; maximum injection time, automated). PRM data used for modeling were acquired with a 4 Da isolation window on the average of the M0 and 2HM3 (or 2HM6 for peptides with 2 leucines) (Supplemental Table 3).

For tracer enrichment studies, peptide stocks (Supplemental Figure 1) were diluted as follows: 1/50 for alpha0 and alpha1, 1/100 to 1/200 for alpha2 and alpha3, and 1/10 for prebeta, with injection volumes ranging from 2 to 6 μL until tracer could be detected at the earliest time point. The same dilution and injection volumes were used for each time point per fraction.

### Spectral processing and PRM library

The DDA spectra were queried against the Human UniProt database (downloaded August 1, 2014) using the HT-SEQUEST search algorithm, via the Proteome Discoverer (PD) Package (version 2.1, Thermo Scientific), using a 10 ppm tolerance window in the MS1 search space and a 0.02 Da fragment tolerance window for HCD data. Methionine oxidation was set as a variable modification. The peptide false discovery rate of 1% was calculated using Percolator provided by PD. Peptides assigned to a given protein group, and not present in any other protein group, were considered unique. For the PRM spectral library, 1 to 3 leucine-containing peptides per protein were used to monitor D3-Leu enrichment. APOE, APOA1, and LCAT peptides were chosen based on their consistency across our previous studies (13, 14, 29); and the 1 peptide for each CETP and PLTP (2 were evaluated per protein) was chosen based on consistent enrichment across 3 participants (Supplemental Table 3).

### Peptide enrichment quantification

We employed our published software, XPI (v.1.3) (29), for the quantification of APOA1 and APOE PRM ions. The mass difference of deuterated leucine labeling was 3.01883025 Da (Supplemental Table 3). The mass tolerance window in XPI for the identification of PRM ions was 0.01 Da. Given the very low tracer signals for the newly reported PLTP, CETP, and LCAT data, we manually quantified PRM ions using the extracted ion chromatogram method (XCalibur Software, Thermo Fisher Scientific). We considered only the 2HM3 ions for tracer even if 2 leucines were in a given fragment since the probability of observing in 2 leucines is less than in 1. We calculated the tracer enrichment as M3 / (M0+M3), reporting it as percentage enrichment for the enrichment plots.

### Absolute quantification of peptides

Cell-free synthesized peptide standards were used to quantify the pool size of APOA1, APOE, LCAT, CETP, and PLTP across the 5 HDL sizes and in total HDL (sum of 5 HDL sizes) in the 6 participants. Each protein was quantified using the following peptide standards: APOA1 (THLAPYSDEL[R-labeled]), APOE (LGPLVEQG[R-labeled]), LCAT (SSGLVSNAPGVQI[R-labeled]), CETP (ASYPDITGE[K-labeled]), and PLTP (AVEPQLQEEE[R-labeled]) (New England Peptides, NEP, Supplemental Table 2). The peptides were quantified by the absolute amino acid method (NEP). Arginines were labeled with 13C8,15N2 and lysines with 13C6,15N2. Peptide standards were chosen based on the following: 1) fully cleaved, 2) devoid of methionines and cysteines, 3) highest ionization/signal intensity relative to the other observed peptides passing criteria 1 and 2, and 4) not reported to be posttranslationally modified (https://www.uniprot.org). As a consequence of these criteria, the peptides used to monitor enrichment in CETP and PLTP were not used for absolute quantification.

We established the appropriate spike-in amount for the peptides by determining the linear range of ionization (AUC of M0) for both the standard and sample-derived peptides. Due to the large dynamic range of the sample peptides, we used 2 spiking mixtures. The first mixture contained a final on-column amount of 100 fmol of APOA1 peptide, 10 fmol of APOE peptide, and 1 fmol of LCAT, CETP, and PLTP peptides. The second mixture contained the same peptides at a 10-fold lower on-column concentration. Peptide abundance was quantified from 2 injection replicates of the 2- and 4-hour time points (diluted 1/100 from the alpha and 1/50 from the prebeta peptide stocks) for the 2 spiking mixtures (8 total quantification replicates per sample). The peptide mixtures were analyzed using the Lumos, using an MS1 scan alone, scan range from 420 to 720 *m/z* at a resolution of 240 K, that was sufficient to capture all sample and standard-derived peptide pairs (Supplemental Table 2). Skyline (https://skyline.gs.washington.edu, ref. 58) was used for quantification of the AUCs.

### HDL protein pool sizes

The pool size (total milligrams of protein in plasma) of APOA1, APOE, CETP, PLTP, and LCAT on the 5 HDL sizes was determined by first converting the fmol on-column (average of 8 replicates) of each protein per size fraction to milligrams of protein per 1 mL of plasma (13, 14). To determine the amount of sample loss during preparation, the mg/mL of APOA1 per size fraction were summed to get an estimated total APOA1 concentration. This estimated total APOA1 concentration was then compared to the total plasma APOA1 concentration (average of 2- and 4-hour time points, Supplemental Table 1), as determined by enzyme-linked immunosorbent assay (ELISA) using anti-APOA1 antibodies (Academy Biomedical catalog 11A-G2b for coating antibody, catalog 11B-G2b for detection antibody). The sample loss correction factor was calculated by dividing the ELISA total APOA1 concentration by the estimated total APOA1 concentration and determined to be 41 (±5). Assuming that sample loss was similar for all size fractions, the mg/mL estimated protein concentrations for each size fraction were then multiplied by the correction factor to determine the mg/mL concentration of each protein in each HDL size fraction. The mg/mL protein concentrations per size were then multiplied by the total plasma volume to determine the protein pool size per HDL size fraction. Plasma volume for each participant was calculated by the following formula (59): plasma volume (dL) = (ideal body weight in kg × 0.44) + (excess body weight in kg × 0.1).

### Variance component analysis and compartmental modeling

Both are detailed in the Supplemental Methods.

### Statistics

All statistical analyses independent of SAAM II were done in R or Microsoft Excel. Scatter plots were used for plotting tracer enrichment. The geometric smoothing function using the local regression (loess) method (R) was applied to PLTP and CETP plots (Figure 2B). The variances reported for PLTP and CETP (Figure 2C) are from all enrichment data points plotted in Figure 2B. The variances of enrichment for APOA1 were calculated from 6 APOA1 fragment ions at each time point. The average was then taken across all time points per HDL fraction. The *P* values calculated from a 1-sided Student’s *t* test assuming unequal variance verified that the sample variance of the Q Exactive was greater than the sample variance of the Lumos for alpha3 and prebeta (Supplemental Figure 3C, less than 0.05). The difference in the average of variance between the Q Exactive and Lumos ranged from –1.04E-06 to –5.79E-07. Box-and-whisker plots were used to display the interquartile and overall range of enrichment data detected by each instrument at each time point. Using the data generated by compartmental modeling, residual plots for each enrichment time point for each HDL size was calculated by: residual = enrichment data point – enrichment model fit. Results are presented as median or mean (SD) unless otherwise specified. Figures were compiled in Microsoft PowerPoint or Adobe Photoshop.

### Study approval

The participants in the study gave written informed consent. This study was approved by the Institutional Review Board of Brigham and Women’s Hospital and Harvard T.H. Chan School of Public Health (IRB 2010P001743/BWH).

## Author contributions

SAS conceived the technological advancement, coordinated mass spectrometry acquisition and enrichment calculations by coauthors, and thus is listed first. SAS and ABA wrote the manuscript. ABA interpreted enrichment data using compartmental modeling analyses. SAS and HH acquired and analyzed mass spectrometric data. ABA prepared HDL samples and performed compartmental modeling. LHL and AR analyzed mass spectrometric data. FMS and MA supported the study and edited the manuscript.

## Supplementary Material

Supplemental data

Supplemental Tables 1-5

## Version 1 12/22/2020

In-Press Preview

## Version 2 02/08/2021

Electronic publication

## References

[1] Singh SA, Aikawa M (2017). Unbiased and targeted mass spectrometry for the HDL proteome. Curr Opin Lipidol.

[2] Chan DC (2004). Lipoprotein transport in the metabolic syndrome: methodological aspects of stable isotope kinetic studies. Clin Sci (Lond).

[3] Burnett JR, Barrett PH (2002). Apolipoprotein B metabolism: tracer kinetics, models, and metabolic studies. Crit Rev Clin Lab Sci.

[4] Miller GJ, Miller NE (1975). Plasma-high-density-lipoprotein concentration and development of ischaemic heart-disease. Lancet.

[5] Castelli WP (1977). HDL cholesterol and other lipids in coronary heart disease. The cooperative lipoprotein phenotyping study. Circulation.

[6] Gordon T (1977). High density lipoprotein as a protective factor against coronary heart disease. The Framingham Study. Am J Med.

[7] Tariq SM (2014). HDL hypothesis: where do we stand now?. Curr Atheroscler Rep.

[8] Tuteja S, Rader DJ (2014). Dyslipidaemia: cardiovascular prevention—end of the road for niacin?. Nat Rev Endocrinol.

[9] Tall AR, Rader DJ (2018). Trials and tribulations of CETP inhibitors. Circ Res.

[10] Wang D (2014). Characterization of human plasma proteome dynamics using deuterium oxide. Proteomics Clin Appl.

[11] Price JC (2012). Measurement of human plasma proteome dynamics with (2)H(2)O and liquid chromatography tandem mass spectrometry. Anal Biochem.

[12] Bateman RJ (2006). Human amyloid-beta synthesis and clearance rates as measured in cerebrospinal fluid in vivo. Nat Med.

[13] Singh SA (2016). Multiple apolipoprotein kinetics measured in human HDL by high-resolution/accurate mass parallel reaction monitoring. J Lipid Res.

[14] Andraski AB (2019). Effects of replacing dietary monounsaturated fat with carbohydrate on HDL (High-Density Lipoprotein) protein metabolism and proteome composition in humans. Arterioscler Thromb Vasc Biol.

[15] Rosenson RS (2011). HDL measures, particle heterogeneity, proposed nomenclature, and relation to atherosclerotic cardiovascular events. Clin Chem.

[16] Kontush A (2013). Unraveling the complexities of the HDL lipidome. J Lipid Res.

[17] Glomset JA (1962). The mechanism of the plasma cholesterol esterification reaction: plasma fatty acid transferase. Biochim Biophys Acta.

[18] Glomset JA (1970). Physiological role of lecithin-cholesterol acyltransferase. Am J Clin Nutr.

[19] Tollefson JH (1988). Isolation and characterization of a phospholipid transfer protein (LTP-II) from human plasma. J Lipid Res.

[20] Korhonen A (1998). Remodeling of HDL by phospholipid transfer protein: demonstration of particle fusion by 1H NMR spectroscopy. Biochem Biophys Res Commun.

[21] Lusa S (1996). The mechanism of human plasma phospholipid transfer protein-induced enlargement of high-density lipoprotein particles: evidence for particle fusion. Biochem J.

[22] Tall AR (1986). Plasma lipid transfer proteins. J Lipid Res.

[23] Bruce C (1998). Plasma lipid transfer proteins, high-density lipoproteins, and reverse cholesterol transport. Annu Rev Nutr.

[24] Barter PJ (2003). Cholesteryl ester transfer protein: a novel target for raising HDL and inhibiting atherosclerosis. Arterioscler Thromb Vasc Biol.

[25] Feghaly JJ, Mooradian AD (2020). The rise and fall “ing” of the HDL hypothesis. Drugs.

[26] Freeman LA (2020). Novel lecithin: cholesterol acyltransferase-based therapeutic approaches. Curr Opin Lipidol.

[27] Mendivil CO (2016). Novel pathways of apolipoprotein a-i metabolism in high-density lipoprotein of different sizes in humans. Arterioscler Thromb Vasc Biol.

[28] Reyes-Soffer G (2016). Cholesteryl ester transfer protein inhibition with anacetrapib decreases fractional clearance rates of high-density lipoprotein apolipoprotein A-I and plasma cholesteryl ester transfer protein. Arterioscler Thromb Vasc Biol.

[29] Lee LH (2017). Automation of PRM-dependent D3-Leu tracer enrichment in HDL to study the metabolism of apoA-I, LCAT and other apolipoproteins. Proteomics.

[30] Oka T (2000). Distribution of phospholipid transfer protein in human plasma: presence of two forms of phospholipid transfer protein, one catalytically active and the other inactive. J Lipid Res.

[31] Lassman ME (2014). Practical immunoaffinity-enrichment LC-MS for measuring protein kinetics of low-abundance proteins. Clin Chem.

[32] Lee AY (2012). Measurement of fractional synthetic rates of multiple protein analytes by triple quadrupole mass spectrometry. Clin Chem.

[33] Kaufmann A, Walker S. Coalescence and self-bunching observed in commercial high-resolution mass spectrometry instrumentation. [Published online January 3, 2018]. Rapid Commun Mass Spectrom . 10.1002/rcm.8054 29297948

[34] Levy MJ (2018). Probing the sensitivity of the orbitrap lumos mass spectrometer using a standard reference protein in a complex background. J Proteome Res.

[35] Sacks FM, Jensen MK (2018). From high-density lipoprotein cholesterol to measurements of function: prospects for the development of tests for high-density lipoprotein functionality in cardiovascular disease. Arterioscler Thromb Vasc Biol.

[36] Eliuk S, Makarov A (2015). Evolution of orbitrap mass spectrometry instrumentation. Annu Rev Anal Chem (Palo Alto Calif).

[37] Schilling B (2015). Multiplexed, scheduled, high-resolution parallel reaction monitoring on a full scan QqTOF instrument with integrated data-dependent and targeted mass spectrometric workflows. Anal Chem.

[38] Peterson AC (2012). Parallel reaction monitoring for high resolution and high mass accuracy quantitative, targeted proteomics. Mol Cell Proteomics.

[39] Kujiraoka T (2003). Effects of intravenous apolipoprotein A-I/phosphatidylcholine discs on LCAT, PLTP, and CETP in plasma and peripheral lymph in humans. Arterioscler Thromb Vasc Biol.

[40] Huang R (2011). Apolipoprotein A-I structural organization in high-density lipoproteins isolated from human plasma. Nat Struct Mol Biol.

[41] Jiang XC (2018). Phospholipid transfer protein: its impact on lipoprotein homeostasis and atherosclerosis. J Lipid Res.

[42] Hailman E (1996). Neutralization and transfer of lipopolysaccharide by phospholipid transfer protein. J Biol Chem.

[43] Gautier T (2008). Effect of plasma phospholipid transfer protein deficiency on lethal endotoxemia in mice. J Biol Chem.

[44] Shamburek RD (2016). Familial lecithin:cholesterol acyltransferase deficiency: First-in-human treatment with enzyme replacement. J Clin Lipidol.

[45] Rye KA, Barter PJ (1994). The influence of apolipoproteins on the structure and function of spheroidal, reconstituted high density lipoproteins. J Biol Chem.

[46] Rye KA (1995). The influence of cholesteryl ester transfer protein on the composition, size, and structure of spherical, reconstituted high density lipoproteins. J Biol Chem.

[47] Lagrost L (1990). Role of cholesteryl ester transfer protein (CETP) in the HDL conversion process as evidenced by using anti-CETP monoclonal antibodies. J Lipid Res.

[48] Melchior JT (2017). Apolipoprotein A-II alters the proteome of human lipoproteins and enhances cholesterol efflux from ABCA1. J Lipid Res.

[49] Ronsein GE, Vaisar T (2019). Deepening our understanding of HDL proteome. Expert Rev Proteomics.

[50] Zhang L (2012). Structural basis of transfer between lipoproteins by cholesteryl ester transfer protein. Nat Chem Biol.

[51] Zhang M (2015). HDL surface lipids mediate CETP binding as revealed by electron microscopy and molecular dynamics simulation. Sci Rep.

[52] Lauer ME (2016). Cholesteryl ester transfer between lipoproteins does not require a ternary tunnel complex with CETP. J Struct Biol.

[53] McLean J (1986). Human lecithin-cholesterol acyltransferase gene: complete gene sequence and sites of expression. Nucleic Acids Res.

[54] Hamilton RL (1986). Nascent high density lipoproteins from liver perfusates of orotic acid-fed rats. J Lipid Res.

[55] Miller NE (2016). Mechanism and physiologic significance of the suppression of cholesterol esterification in human interstitial fluid. Front Pharmacol.

[56] Hamilton RL (1976). Discoidal bilayer structure of nascent high density lipoproteins from perfused rat liver. J Clin Invest.

[57] Cheung MC (1986). Distribution and localization of lecithin:cholesterol acyltransferase and cholesteryl ester transfer activity in A-I-containing lipoproteins. J Lipid Res.

[58] MacLean B (2010). Skyline: an open source document editor for creating and analyzing targeted proteomics experiments. Bioinformatics.

[59] Nikkilä EA, Kekki M (1972). Plasma triglyceride metabolism in thyroid disease. J Clin Invest.

