# Supplementary material for: Metabolism of PLTP, CETP, and LCAT on multiple HDL sizes using the Orbitrap Fusion Lumos
Source: JCI Insight. 2021 Feb 8;6(3):e143526. doi: 10.1172/jci.insight.143526 (PMC7934878; doi:10.1172/jci.insight.143526)
Supplement: Supplemental data [file jciinsight-6-143526-s092.pdf]

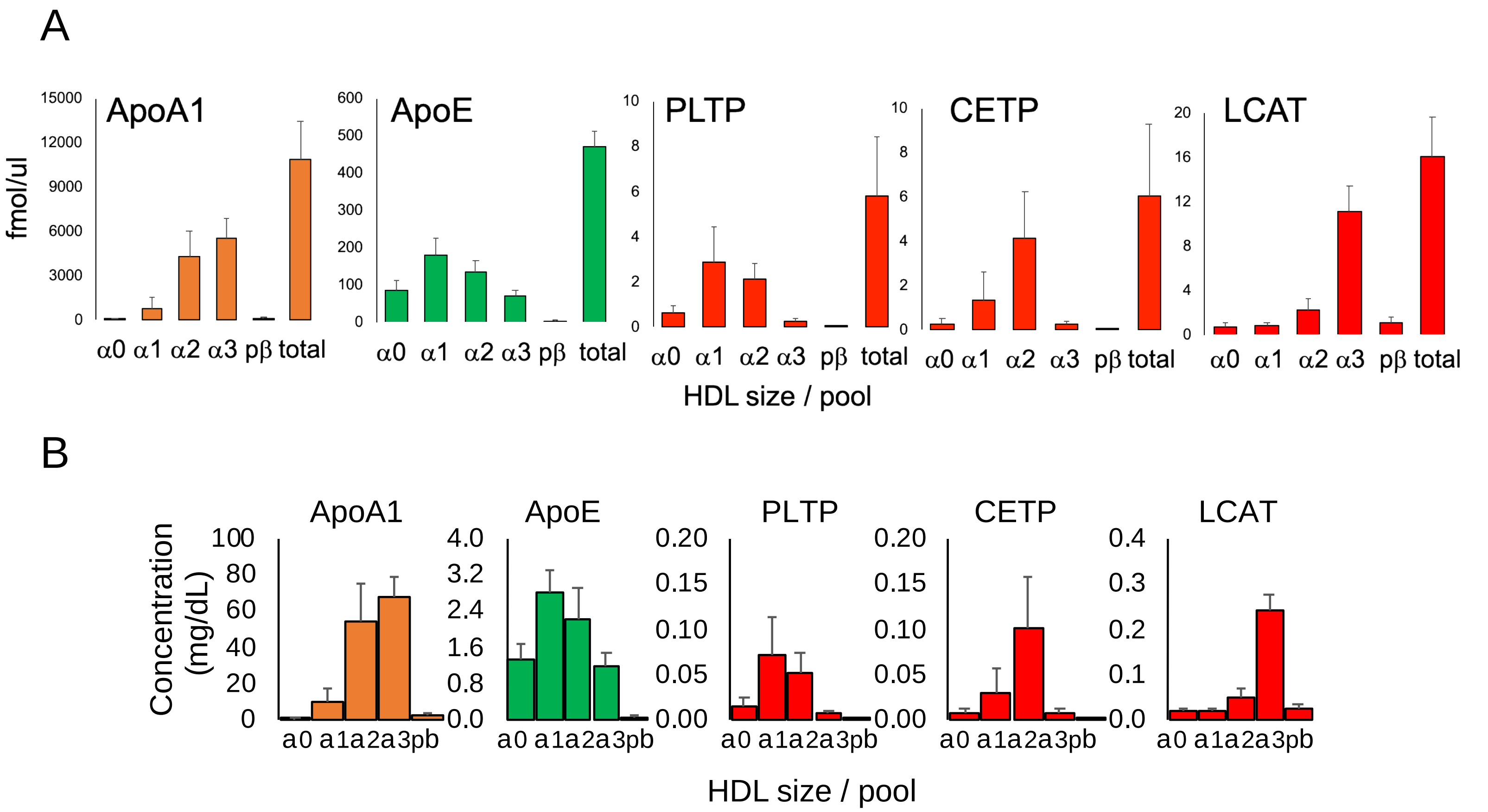

Supplemental Figure 1. Distribution of proteins across HDL sizes. Values are calculated averages from six participants. Error bars are standard deviations. (**A**) fmol/ul peptide stocks obtained from each HDL size, calculated using stable isotope labeled peptides (see Methods, *Absolute quantification of peptides*). (**B**) Total protein pools per HDL size, calculated using stable isotope standards and ELISA (see Methods, *HDL protein pool sizes*).

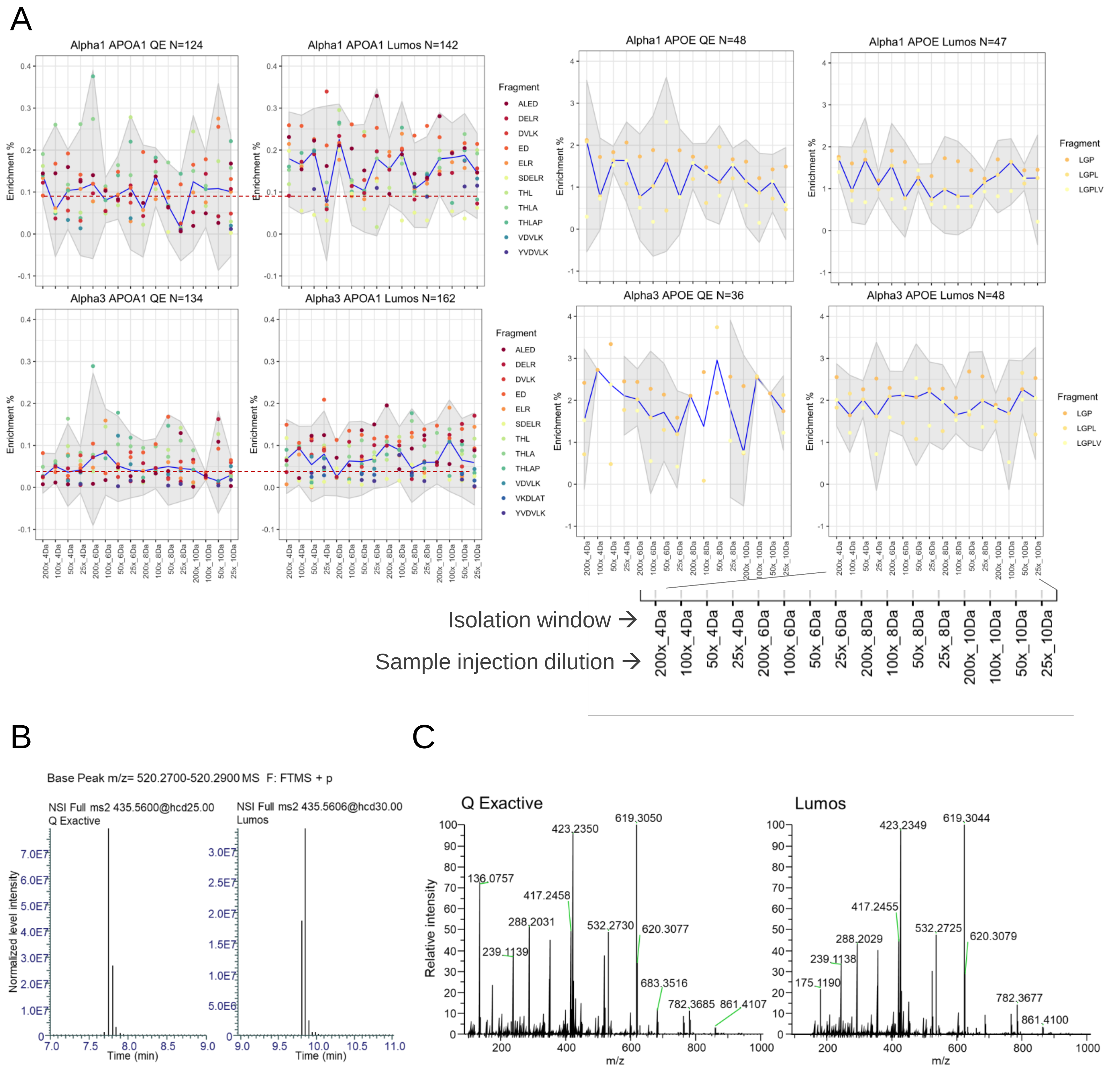

Supplemental Figure 2. The effects of isolation window and injection concentration on enrichment. (**A**) The effects of sample injection dilution and PRM isolation window on D3-Leu enrichment ([2HM3]/[2HM3+M0]), performed on the Q Exactive (R=120K @ *m/z* 200) and Lumos (R=240K @ *m/z* 200) platforms in an unscheduled mode. ApoA1: 3 peptides, 12 PRM ions; ApoE: 1 peptide, 3 PRM ions (Supplemental Table 2). Timepoint, 0.5 hr post D3-Leu bolus from Participant 1. Enrichment is most affected by instrument platform, especially for the slowly metabolizing ApoA1 whose net enrichment is lower on the Q Exactive than on the Lumos (dashed red line). Grey indicates points that fall within the 95% confidence interval using the mean standard deviation (+/-) from the median (blue line). (**B**) Extracted ion chromatogram of the *b5* ion of ApoA1 THLAPYSDELR analyzed for the 0.5 hr timepoint in alpha3. (**C**) The PRM spectra from panel B.

A

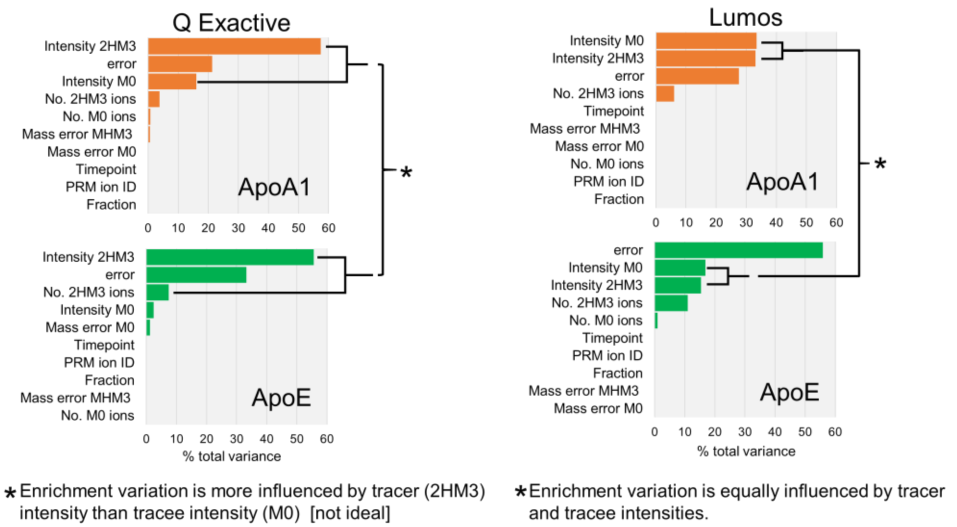

B

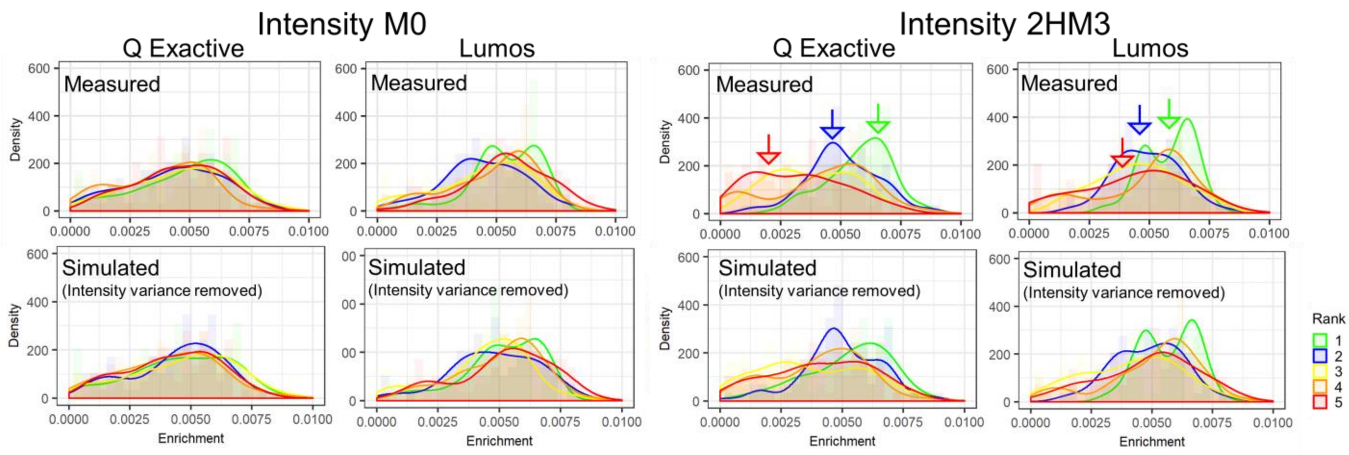

$$y = \frac{I_t}{I_t + I_T}$$

C

	Average of variances		Difference	T-test (one-sided, unequal variance
HDL size	Q Exactive	Lumos	Lumos-Q Exactive	*p*-value
alpha0	2.02E-06	9.84E-07	-1.04E-06	0.1014
alpha1	1.55E-06	1.39E-06	-1.68E-07	0.3469
alpha2	1.86E-06	1.55E-06	-3.12E-07	0.3168
alpha3	1.49E-06	9.08E-07	-5.79E-07	0.0410
prebeta	2.14E-06	6.95E-07	-1.45E-06	0.0001

Supplemental Figure 3. Enrichment variation and compression due to low abundant tracer signal is reduced on the Lumos. (**A**) Variance component analysis summary of ApoA1 and ApoE enrichment across five HDL size fractions (Participant 1). (**B**) Density plots depicting the distribution of tracer enrichment by M0 (tracee) or 2HM3 (tracer) intensity ranks (Rank 1 to 5, top to bottom quintiles, respectively). Data from all five HDL size fractions and timepoints were used (Participant 1). The simulated data were calculated using the indicated formula: For each PRM M0 ion (tracee) in each HDL size fraction, we calculated the average intensity, and assumed that to be the ground truth (steady-state) tracee intensity (I_T). For each timepoint in each HDL size fraction, we calculated the median enrichment (*y*), and assumed that to be the ground truth enrichment profile. We then calculate the "expected/ideal' 2HM3 intensity (I_t). (**C**) A comparison of the 'Average of variances' between the Q Exactive and the Lumos for each HDL size. The 'Difference' between the averages of variance of HDL size shows that the Lumos exhibits less variability.

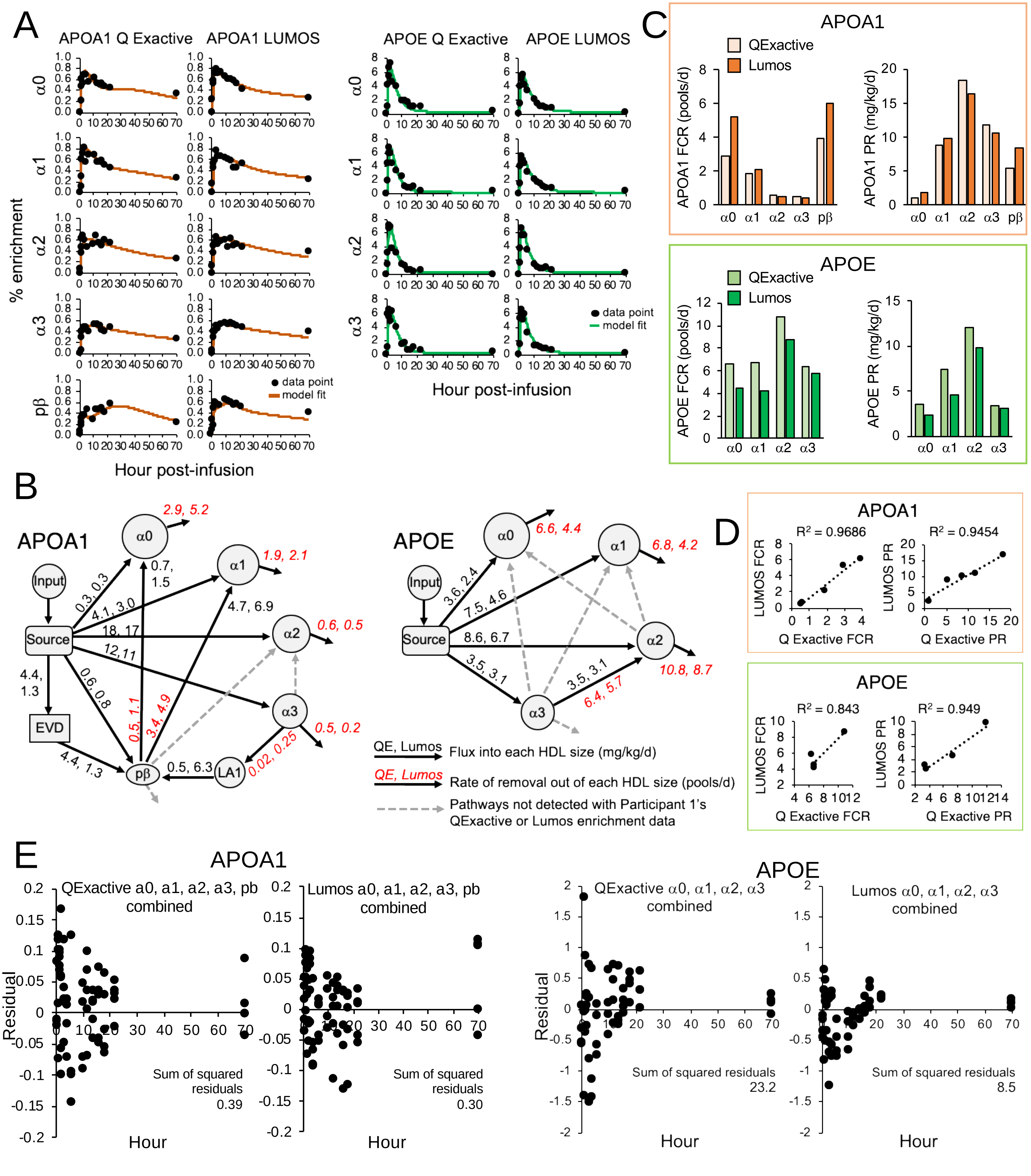

Supplemental Figure 4. An inter-instrument platform comparison of compartmental modeling data. (**A**) APOA1 and APOE enrichment curve fits on each HDL size generated from their respective compartmental models. n=14 time points for Participant 1. (**B**) The compartment models use to determine the FCR and PR of APOA1 and APOE on each HDL size. (**C**) FCR and PR for each protein and instrument on each HDL size fraction. (**D**) Correlation analysis of the metabolic rates generated by each instrument. (**E**) The sum of squared residuals (SSR, enrichment data-enrichment model fit) for each protein, when considering all HDL size data. The SSRs of the QExactive > than those of the Lumos indicating a larger data point spread around the model fits. PR, production rate; FCR, fractional catabolic rate.

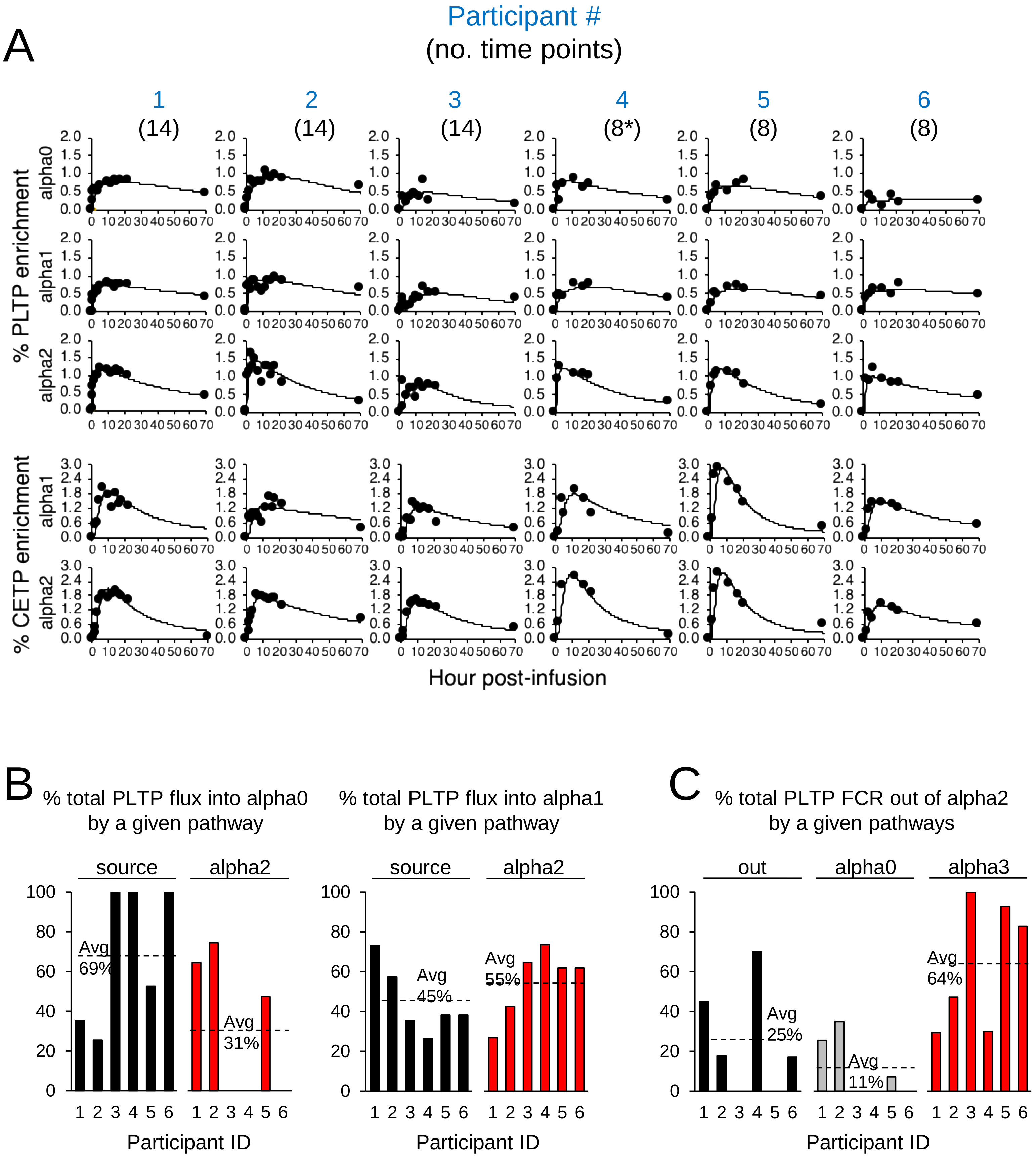

Supplemental Figure 5. PLTP and CETP compartmental modeling data. (**A**) Enrichment curve model fits for PLTP and CETP. (**B**) Contribution of source and alpha2 to %total PLTP flux (**C**). % total PLTP FCR out of alpha2 from the indicated pathways. * Participant 4's alpha2 has n=7 time points

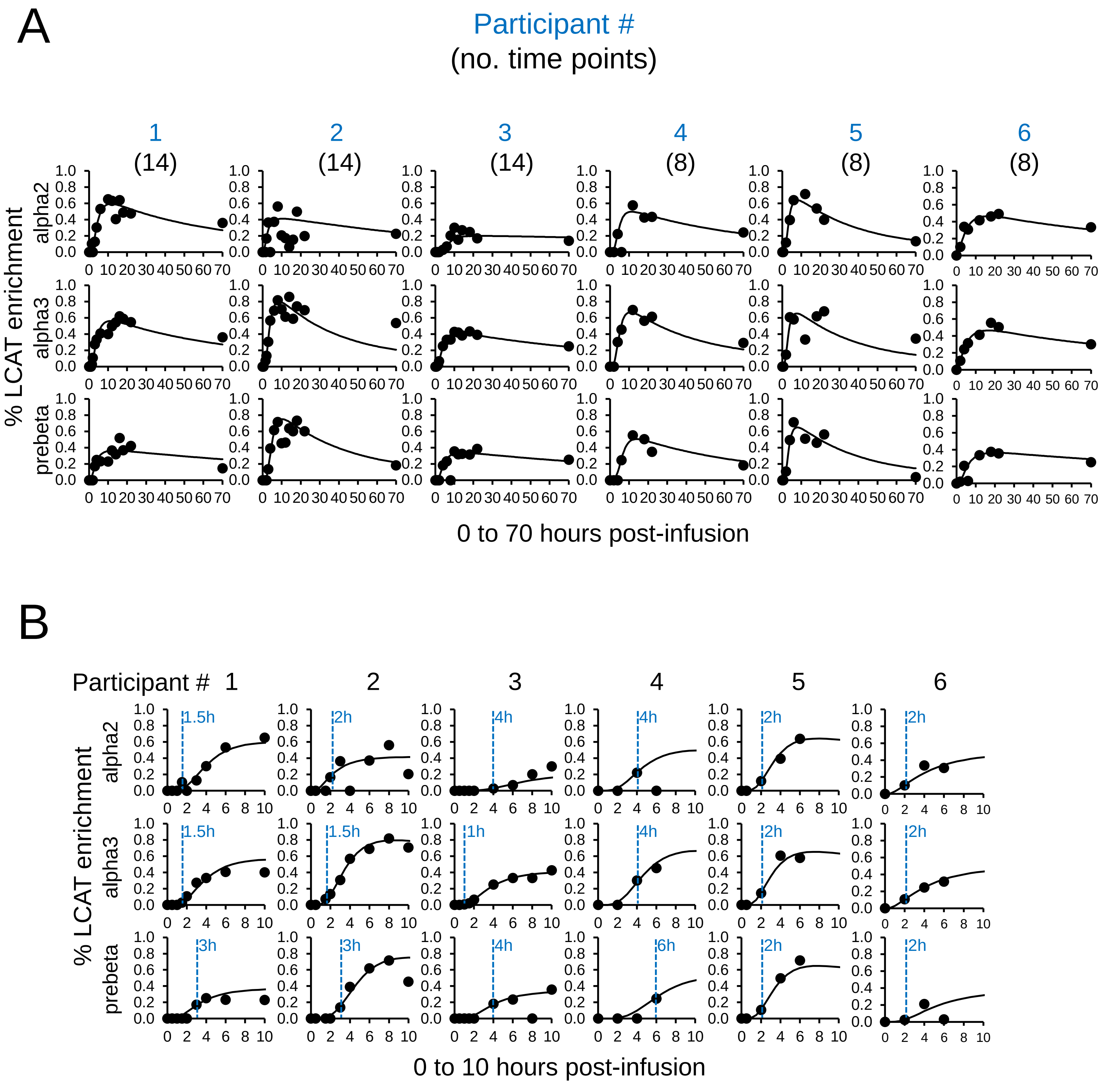

Supplemental Figure 6. **LCAT enrichment curve model fits**. (**A**) Full time course experiment. (**B**) Zoom into the first ten hours of the time course. Dashed blue line and hour indicate the time point in which LCAT tracer was first detected on a given HDL size in plasma per participant. LCAT on prebeta HDL tended to appear in circulation later than LCAT on alpha3 for 4 out of 6 participants.

Metabolism of PLTP, CETP, and LCAT on multiple HDL sizes using the Orbitrap Fusion Lumos

Sasha A. Singh[1]*, Allison B. Andraski[2]*, Hideyuki Higashi[1], Lang Ho Lee[1],
Ashisha Ramsaroop[1], Frank M. Sacks[2,3] and Masanori Aikawa[1,3,4]

SUPPLEMENTAL METHODS

Variance component analysis. The variance component analysis (VCA) was used to estimate the contribution of random effects to the variance of the dependent variable. The VCA is helpful to understand where to focus attention in order to reduce the variance. We employed 'VCA' R package to estimate the contribution of 10 variables to the variance of the dependent variable, the enrichment (https://cran.r-project.org/web/packages/VCA/index.html). For the VCA procedure, we binned continuous variables, for example ion intensity and the number of PRM ions, into five ranks (1 for high and 5 for low). The analyzed variables include, 'rank by heavy and light ion intensity', 'rank by the number of PRM ions of heavy and light ions', 'rank by max delta mass difference of heavy and light ions', 'instrument', 'fraction' and 'ion fragment'. Graphical presentation of the VCA analysis results was done by 'ggplot2' R package.

Compartmental modeling. Compartmental modeling was performed using SAAM II software (The Epsilon Group, http://tegvirginia.com) (1). Although enrichment was monitored for APOA1, APOE, PLTP, CETP, and LCAT across the five HDL sizes, only HDL sizes in which D3-Leu label was consistently detected in all participants were included in the model for each protein: APOA1 in alpha0, alpha1, alpha2, alpha3 and prebeta; APOE in alpha0, alpha1, alpha2 and alpha3; PLTP in alpha0, alpha1, and alpha2; CETP in alpha1 and alpha2; and LCAT in alpha2, alpha3 and prebeta. The APOA1, APOE, and LCAT alpha3 models were developed previously (2, 3). This is the first study to our knowledge to determine the metabolism of LCAT on alpha2 and prebeta, and of CETP and PLTP on HDL.

Each model contains an input, source, and HDL size compartments. The input compartment is the plasma amino acid precursor pool (D3-Leu tracer enrichment in plasma) expressed as a forcing function that drives the appearance of D3-Leu tracer in the model. Each participant's plasma D3-Leu tracer enrichment curve was used for all protein models. The source compartment accounts for the time necessary for D3-Leu-labeled protein to appear on each HDL size in plasma. One to five compartments were added to each model that represented the HDL sizes. The pool size and tracer enrichment data were assigned to each HDL size compartment. Two additional compartments were included in the APOA1 model: 1) delay compartment connecting the source and prebeta. This may represent an extravascular delay (EVD) processing compartment that includes APOA1 prebeta that has been secreted but is outside systemic circulation (2, 3). 2) Compartment

connecting alpha3 and prebeta. This compartment may represent lipidated APOA1 (LA1) that has been released from alpha3 and is used to generate prebeta (2, 3). Three additional delay compartments were included in the LCAT model: delay compartments connecting the source to alpha2, the source to alpha3, and the source to prebeta. These delays were necessary to account for the difference in time of LCAT tracer appearance on the different HDL sizes and across participants. For all models, a direct secretion pathway from the source into each size, or from the source to a delay and then into each HDL size in the case of LCAT, was required for satisfactory fitting. Pathways among the sizes were included in the final model when 1) flux through that pathway was detected in at least two participants' models, and 2) the detected flux improved overall model fits for participants in which the pathway was detected. A removal pathway out of each HDL size was also included for each protein model. This pathway represents the removal of a given protein out of a given HDL size fraction in circulation, such as by hepatic uptake or by protein transfer to a compartment not measured in our study (i.e., the lipoprotein-free fraction or an APOB-containing lipoprotein).

The following kinetic parameters were calculated for 1 participant in the APOA1 and APOE models (participant 1) and for 6 participants in the PLTP, CETP, and LCAT models: 1) fractional catabolic rate (FCR), the fraction of a given plasma protein pool turned over per day was determined for each protein in each HDL size by taking the sum of the rate constants exiting that compartment. 2) Production rate, the amount (mg) of protein produced or transferred into each HDL size/day/kg of body weight. Production rate = FCR (pools/day) x pool size (mg) / body weight (kg). 3) Flux of a protein from the source compartment into each HDL size, and from one HDL size to another (mg/kg/day). 4) Rate of removal (*k*) out of each HDL size by a given pathway (pools/day). Steady-state kinetics was assumed for all proteins.

REFERENCES

1. Barrett PH, Bell BM, Cobelli C, Golde H, Schumitzky A, Vicini P, et al. SAAM II: Simulation, Analysis, and Modeling Software for tracer and pharmacokinetic studies. *Metabolism.* 1998;47(4):484-92.
2. Singh SA, Andraski AB, Pieper B, Goh W, Mendivil CO, Sacks FM, et al. Multiple apolipoprotein kinetics measured in human HDL by high-resolution/accurate mass parallel reaction monitoring. *J Lipid Res.* 2016;57(4):714-28.
3. Andraski AB, Singh SA, Lee LH, Higashi H, Smith N, Zhang B, et al. Effects of Replacing Dietary Monounsaturated Fat With Carbohydrate on HDL (High-Density Lipoprotein) Protein Metabolism and Proteome Composition in Humans. *Arterioscler Thromb Vasc Biol.* 2019:ATVBAHA119312889.
